# DeepMHCI: an anchor position-aware deep interaction model for accurate MHC-I peptide binding affinity prediction

**DOI:** 10.1093/bioinformatics/btad551

**Published:** 2023-09-05

**Authors:** Wei Qu, Ronghui You, Hiroshi Mamitsuka, Shanfeng Zhu

**Affiliations:** Institute of Science and Technology for Brain-Inspired Intelligence and MOE Frontiers Center for Brain Science, Fudan University, Shanghai 200433, China; Institute of Science and Technology for Brain-Inspired Intelligence and MOE Frontiers Center for Brain Science, Fudan University, Shanghai 200433, China; Bioinformatics Center, Institute for Chemical Research, Kyoto University, Uji, Kyoto Prefecture 611-0011, Japan; Department of Computer Science, Aalto University, 00076 Espoo, Finland; Institute of Science and Technology for Brain-Inspired Intelligence and MOE Frontiers Center for Brain Science, Fudan University, Shanghai 200433, China; Shanghai Qi Zhi Institute, Shanghai 200030, China; Key Laboratory of Computational Neuroscience and Brain-Inspired Intelligence, Fudan University, Ministry of Education, Shanghai 200433, China; Shanghai Key Lab of Intelligent Information Processing and Shanghai Institute of Artificial Intelligence Algorithm, Fudan University, Shanghai 200433, China; Zhangjiang Fudan International Innovation Center, Shanghai 200433, China

## Abstract

**Motivation:**

Computationally predicting major histocompatibility complex class I (MHC-I) peptide binding affinity is an important problem in immunological bioinformatics, which is also crucial for the identification of neoantigens for personalized therapeutic cancer vaccines. Recent cutting-edge deep learning-based methods for this problem cannot achieve satisfactory performance, especially for non-9-mer peptides. This is because such methods generate the input by simply concatenating the two given sequences: a peptide and (the pseudo sequence of) an MHC class I molecule, which cannot precisely capture the anchor positions of the MHC binding motif for the peptides with variable lengths. We thus developed an anchor position-aware and high-performance deep model, DeepMHCI, with a position-wise gated layer and a residual binding interaction convolution layer. This allows the model to control the information flow in peptides to be aware of anchor positions and model the interactions between peptides and the MHC pseudo (binding) sequence directly with multiple convolutional kernels.

**Results:**

The performance of DeepMHCI has been thoroughly validated by extensive experiments on four benchmark datasets under various settings, such as 5-fold cross-validation, validation with the independent testing set, external HPV vaccine identification, and external CD8+ epitope identification. Experimental results with visualization of binding motifs demonstrate that DeepMHCI outperformed all competing methods, especially on non-9-mer peptides binding prediction.

**Availability and implementation:**

DeepMHCI is publicly available at https://github.com/ZhuLab-Fudan/DeepMHCI.

## 1 Introduction

Major histocompatibility complex (MHC) molecules are essential in the T-cell-mediated adaptive immune response (Janeway *et al.* 2005). In a general procedure of antigen processing, antigens are first derived from protein cleavage in the proteasome and then transported as a peptide by an associated transporter and subsequently recognized and bound to specific MHC molecules to produce a peptide–MHC complex (pMHC). Finally, the pMHC is delivered to the surface of nucleated cells and recognized by specific T-cell receptors on the outer cell surface, which activates the subsequent adaptive immune response. Accurate identification of MHC-binding peptides is thus not only essential to elucidate the mechanisms of MHC-peptide binding but also to facilitate the design of peptide-based vaccines for cancer immunotherapies. Great efforts have been made to develop computational methods for MHC-peptide binding prediction, which have been utilized to analyze binding motifs and select promising candidate peptides to conduct biochemical experiments for verification (Ott *et al.* 2017).

MHC molecules are generally divided into two types: MHC class I (MHC-I) and MHC class II (MHC-II). MHC-I and MHC-II molecules play different roles in the adaptive immune response (Janeway *et al.* 2005). MHC-II molecules bind a longer length of peptides from exogenous antigens with specific binding cores. In contrast, the binding groove is closed at both ends in MHC-I molecules leading to a size restriction of the bound peptides (Janeway *et al.* 2005). In general, MHC-I molecules typically bind peptides from endogenous antigens ranging from 8 to 12 in length with several indeterminate anchor residues, which are key to the peptide fitting into pockets inside the groove of the MHC-I molecules and mainly determine the binding affinity. In this study, we focus on MHC-I molecules, aiming at developing insight into interactions of MHC-I molecules and peptides covering all common peptide lengths.

Accurate prediction for the interaction between MHC-I molecules with peptides is a challenging task. The challenges come from three sides: MHC-I molecules, peptides, and their interaction. For the MHC-I molecule side, MHC-I molecules are highly polymorphic and highly specific: each of thousands of MHC-I molecules has its own binding specificity. For the peptide side, due to the size restriction from the binding groove, anchor residue positions may shift in peptides with different lengths (Nielsen and Andreatta 2016), resulting in two issues: the variety in length and the consequent identification of anchor residues. For the interaction, given a pair of an MHC-I molecule and a peptide, the underlying hypothesis is that the linear peptide is wrapped by an MHC molecule groove and the side chains of the amino acids forming the groove pocket point to the short peptide (Anjanappa *et al.* 2020). The main issue is how to introduce this prior knowledge and construct effective interactions between MHC-I molecules and peptides to realize the algorithm’s perception of highly specific MHC-I binding motifs under different peptide lengths.

Computational methods for MHC-I peptide binding prediction have two categories: allele-specific and pan-specific (Zhang *et al.* 2012). In the allele-specific methods, a model is trained for each MHC molecule individually resulting in limited generalization to unseen molecules. (Andreatta and Nielsen 2016, O’Donnell *et al.* 2018, Mei *et al.* 2021). In contrast, pan-specific methods allow to integrate the information of MHC molecules and peptides in one model, which is beneficial for learning the binding specificity of all MHC molecules simultaneously (Nielsen *et al.* 2007a, Zhang *et al.* 2012), solving the above MHC-I molecule issue mostly. However, the issues for the peptide and the interaction sides are unsolved. Most methods obtain binding assay data from the immune epitope database and analysis resource (IEDB) (Vita *et al.* 2019), in which peptide lengths are highly biased towards nine (Jurtz *et al.* 2017). Many algorithms have been designed only for nine amino acids of peptides (9-mers). For peptides with a variable length other than nine, the most established method is NetMHCpan (the latest version for MHC-I peptide binding prediction without data from mass spectrometry assay is NetMHCpan-3.0; Nielsen and Andreatta 2016). NetMHCpan uses a collate function between input peptides and output binding affinity: amino acid deletions or insertions are used to transform a given peptide to 9-mer subsequences, which are the fixed sized input of Artificial Neural Network (ANN), and the binding affinity of the peptide is given by taking the maximum output score of the generated 9-mer sub-sequences. This collate function inevitably causes information loss. Another problem is to simply concatenate two vectors [peptide (9-mer) and MHC-I molecule] for modeling the interaction due to the fixed sized input limitation of ANN.

Recently, advanced machine learning techniques are proposed for MHC-I peptide binding prediction, such as differential boundary tree (DBT), convolutional neural network (CNN), attention mechanism and Transformer. Five representative methods (all are pan-specific) are DeepLigand-BA (Zeng and Gifford 2019), DeepAttentionPan (Jin *et al.* 2021), ACME (Hu *et al.* 2019), DBTpred (Feng *et al.* 2021), and TransPHLA (Chu *et al.* 2022). However, for the peptide side issue, most methods use so-called padding operations for peptide sequences of different lengths without identifying anchor positions and fail to capture the binding motif of MHC-I molecules. Also, the common strategy for the interaction is still to concatenate the vector representations (of MHC-I molecule and peptide) as input. Ignoring the direct interaction between peptides and MHC-I molecules results in unsatisfactory performance on non-9-mer peptides.

**Figure 1.**
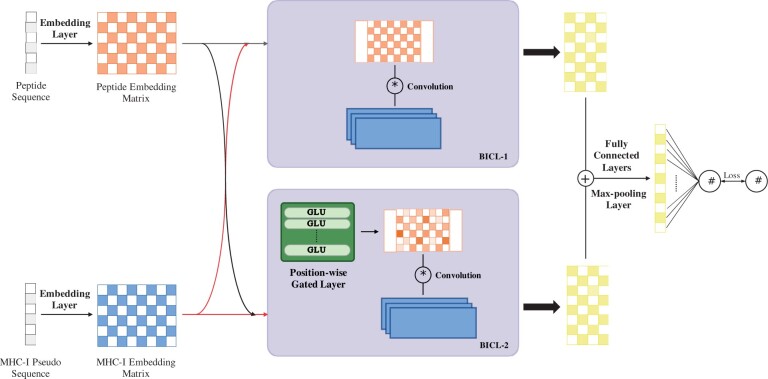
The architecture of DeepMHCI. The arrows from MHC-I embedding matrix to BICL-1 and BICL-2 are the processes of generating kernels from MHC-I embedding matrix.

In this work, we propose a deep learning-based method, DeepMHCI, for predicting MHC-I peptide binding affinity by incorporating biological knowledge into the model design (Fig. 1 for the entire architecture). In DeepMHCI, both the peptide and interaction side issues are simultaneously considered by focusing on two points: (i) anchor position awareness in peptides under different lengths and (ii) direct interaction between a peptide and an MHC-I molecule. Specifically, DeepMHCI controls the information on peptide amino acids directly in terms of length variation, guaranteeing anchor position awareness. Furthermore, an updated residual binding interaction convolution layer (You *et al.* 2022) is applied to model the interaction directly, which allows to consider of the binding (interaction) patterns.

The performance of DeepMHCI was thoroughly validated by extensive experiments on four benchmark datasets under various settings, such as 5-fold cross-validation (5-fold CV), independent testing set verification and two types of external datasets: (i) binding data of peptides derived from HPV16 E6 and E7 proteins and (ii) CD8+ epitopes from IEDB. We compared the predictive performance of DeepMHCI with five state-of-the-art pan-specific methods: NetMHCpan-3.0, DeepLigand-BA, DeepAttentionPan, TransPHLA, and DBTpred. All competing methods were retrained on the same benchmark if possible.

Experimental results demonstrate that DeepMHCI outperformed all competing methods in all experiments, especially for peptides with non-9-mer lengths. For example, DeepMHCI achieved an average AUC of 0.751 in the HPV dataset under 11-mer, which was 9.0% and 13.6% higher than NetMHCpan-3.0 (0.689) and TransPHLA (0.661), respectively. We also verified the performance advantage and interpretability of DeepMHCI in identifying binding motifs of MHC-I molecules under different peptide lengths.

## 2 Materials and methods

### 2.1 Problem formulation

Given a pair of peptide sequence *P* and MHC-I molecule sequence *Q*, the task is a regression problem to predict the binding affinity $\hat{y}$, which is mainly determined by the amino acids bound to the binding groove of the MHC-I molecule in the peptide sequence, i.e. the anchor residues. In addition, it has been found that the binding grooves used by MHC molecules vary when peptides are bound at different lengths. Here we use 34-length pseudo sequence Q′ extracted from the source protein sequence as the representation of MHC-I molecules, which was proposed by NetMHCpan (Nielsen and Andreatta 2016), and also used by DeepLigand-BA, TransPHLA, and DBTpred.

### 2.2 Overview

The basic idea of DeepMHCI is (i) to use a gated mechanism in deep learning to control the expression of amino acids in peptides at different loci at different lengths in a fine-grained manner to sense the anchor position and (ii) to use our proposed residual binding interaction convolution layer (ResBICL) to obtain the representation of binding interaction and promote it to a general residual-connection fashion. (iii) We then use fully connected layers and a max-pooling layer to extract the representation vector and (iv) we use an output layer to obtain the predicted binding affinity. Figure 1 shows the architecture of DeepMHCI.

### 2.3 Input layer

Protein sequences comprised a linear chain of amino acids that can be treated as sequences of tokens. The embedding layer as a dictionary contains 20 standard amino acids with one special character 0 for padding, and each key corresponds to a *d*-dim learnable vector for representation. Given a pair of peptide sequence *P* and MHC-I molecule pseudo sequence Q′, the output matrix of peptide and MHC-I molecule from the embedding layer can be defined as $X\in R^{L\times d}$ and $Y\in R^{34\times d}$, respectively.

### 2.4 Position-wise gated layer

We use a position-wise gated layer to control the bandwidth of the information flow of each amino acid. The gated linear units (GLU) (Dauphin *et al.* 2017) are used as the basic component, which is applied to each position separately and identically. For amino acid vectors at each site in the peptide embedding matrix, $x_{i}\in R^{d},i=1,2,\ldots ,L$, the GLU then takes the form:

(1) $$GLU_{i}(x_{i})=\sigma (W_{i}x_{i}+b_{i})⊙(V_{i}x_{i}+c_{i}),$$

where σ(.) is the sigmoid activation function, $W_{i}\in R^{d\times d},\;V_{i}\in R^{d\times d},\;b_{i}\in R^{d}$, and $c_{i}\in R^{d}$ are learned parameters and ⊙ is the element-wise Hadamard product. GLUs allow the model to control the extent to which the position-wise gated layer contributes to the original peptide embedding matrix **X**, potentially skipping an amino acid entirely if necessary. The first term σ(Wx+b) of each GLU is utilized to control the amino acid expression at the corresponding position and the output of each GLU can be concatenated into matrix $\bar{X}\in R^{L\times d}$, which displays the new representation of the peptide embedding matrix over the gated layer.

### 2.5 Residual binding interaction convolutional layer

We use a novel binding interaction convolutional layer (BICL) to learn the representation of the interactions between the MHC-I embedding matrix and peptide embedding matrix. Traditional convolution kernels are initialized randomly and cannot compute interactions directly or discriminate different MHC-I molecules binding preference effectively. To address these issues, we use a learnable weight matrix $U_{i}\in R^{k\times 34}$ with the MHC-I embedding matrix **Y** to generate the *i*-th kernel $K_{i}^{k}=f(U_{i}Y)$, where f(.) is the activation function, *k* is the kernel size. Using the convolution kernel $K_{i}^{k}$ and $\bar{X}$, the interaction can be computed as follows:

(2) $$C_{i,j}^{k}=f(K_{i}^{k}*{\bar{X}}_{j:j+k-1}+b_{i}^{k}),$$

where $b_{i}^{k}\in R$ is the bias. Therefore, we obtain the representation matrix $C^{k}\in R^{H^{k}\times (L-k+1)}$, where *H^k^* is the number of kernels. In the implementation, we set *k *=* *9, 11, 13 to cover general peptide lengths and obtain interaction information from perspectives of different lengths. Note that the padding character is added to both side of **X** to ensure the same output shape when kernel size *k* is larger than 9. The final output presentation matrix of one BICL is the concatenation of $C^{k}$, written as $\bar{C}\in R^{d(0)\times (L-9+1)}$, where $d(0)=\sum_{k=9,11,13}H^{k}$.

As the output of BICL is the representation of binding interaction which cannot be fed as its input, to reserve the original information from the peptide sequence, two independent BICLs are used as follows and obtain the final output of the ResBICL:

(3) $$C^{\left(0\right)}=f(BICL_{1}(X,Y\;|\;\theta_{1})+BICL_{2}(\bar{X},Y\;|\;\theta_{2})),$$

where f(.) is the activation function, $\theta_{1}$ and $\theta_{2}$ are the parameters of $BICL_{1}$ and $BICL_{2}$, respectively. To avoid overfitting, only one ResBICL is applied to form a shallow network.

### 2.6 Fully connected layer and output layer

In addition to ResBICL, *N* fully connected layers are used to extract better representation:

(4) $$C^{\left(n\right)}=f(W^{\left(n\right)}C^{\left(n-1\right)}+b^{\left(n\right)}),\;\;\;n=1,2,\ldots ,N,$$

where $W^{\left(n\right)}\in R^{d(n)\times d(n-1)},\;b^{\left(n\right)}\in R^{d(n)},\;C^{\left(n\right)}\in R^{d(n)\times (L-9+1)}$ are weights, bias, and output of the *n*-th fully connected layer. We then utilize a max-pooling layer to obtain the final representation vector $g\in R^{d(N)}$:

(5) $$g_{i}=\max\{C_{i,1}^{\left(N\right)},C_{i,2}^{\left(N\right)},\ldots ,C_{i,L-9+1}^{\left(N\right)}\}.$$

The output layer is also a fully connected layer projecting the final representation vector **g** to the predicted binding affinity $\hat{y}\in [0,1]$ with sigmoid function:

(6) $$\hat{y}=\sigma (w^{\left(o\right)}\cdot g+b^{\left(o\right)}),$$

where $w^{\left(o\right)}\in R^{d(N)}$ and $b^{\left(o\right)}\in R$ are weights and bias, respectively. For solving this regression task, our loss function is the mean square error. In the implementation, we train 10 times of 5-fold CV with different random initialization and use the average scores over 50 models as the final prediction to minimize the variance when evaluating test sets.

## 3 Experiments

### 3.1 Datasets

We use four publicly available benchmark datasets (BD2017, ID2022, HPV2019, and EP2017) to train and evaluate DeepMHCI and competing methods: BD2017 for MHC-peptide binding affinities and training models, ID2022 for MHC-peptide binding affinities and classification serving as an independent dataset, HPV2019 and EP2017 serving as the external dataset for HPV vaccine identification and CD8+ epitopes prediction, respectively.

BD2017: it contains 185 985 peptide-MHC binding affinity measurements covering 153 different MHC-I molecules, including 7 BoLA, 1 Gogo, 7 H-2, 104 HLA, 19 Mamu, 11 Patr, and 4 SLA molecules. BD2017 was compiled from IEDB (Vita *et al.* 2019) for training NetMHCpan-3.0 (Nielsen and Andreatta 2016). Twenty-five random natural peptides for each of lengths 8, 9, 10, and 11 as artificial negatives for each allele were introduced when training and excluded from all evaluations. The original experimentally obtained IC50-binding value of each data point was transformed into binding affinity with the range of [0,1] by 1 − log(IC_50_nM)/log(50 000). BD2017 has already provided a strict 5-fold CV split which ensures that no identical 8mer segment was shared between partition as possible. Table 1 shows a summary of BD2017.

**Table 1.** Summary statistics of BD2017.

Allele	No. peptides	No. binders	No. MHCs
BoLA	1264	610	7
Gogo	15	6	1
H-2	9771	2716	7
HLA	156 818	40 841	104
Mamu	14 018	4969	19
Patr	3710	1062	11
SLA	389	201	4
Total	185 985	50 405	153

ID2022: it is a set of automated benchmark datasets that were compiled from IEDB for evaluating different MHC-I binding prediction methods (Trolle *et al.* 2015). We generated an independent test dataset by collecting all benchmarks from 2019 to 2022 and filtered those with over 80% positive binders to ensure a high-quality dataset. Sixty-four benchmarks with a total of 1376 peptides covering 26 MHC molecules were finally identified (see Supplementary material for the benchmark IDs).

HPV2019: a recent study (Bonsack *et al.* 2019) presented an independent experimental binding dataset that was generated in the context of a project on therapeutic HPV16 vaccine design. All peptides were derived from the HPV16 proteins E6 and E7 and have binary labels (binder/nonbinder). In total, HPV2019 consists of 734 8-11-mer peptides covering seven HLA molecules.

EP2017: EP2017 is established in NetMHCpan-4.0 (Jurtz *et al.* 2017) by downloading epitopes from IEDB with restrictions. The final dataset contains 1660 entries and all epitope lengths range from 8-mer to 13-mer covering 52 HLA molecules. The epitope in each entry is annotated as the positive and all other subsequences with the same length from the corresponding source protein are annotated as the negative.

Following You *et al.* (2022), we have evaluated the redundancy of the 5-fold CV split of BD2017 by comparing it with a random split. The comparison showed that the 5-fold CV split ensures very low redundancy between any two folds. The detailed results are shown in Supplementary Table S1.

### 3.2 Experimental settings

DeepMHCI used the following hyperparameter values, which were selected by 5-fold CV over BD2017: *L* (the length of input peptides) =15. *d* (dimension of embeddings of amino acids) =16. The numbers of kernels of ResBICL with the kernel sizes of 9, 11, and 13 were 128, 64, and 32, respectively. *N* (number of fully connected layers) =2 and the number of neurons at two layers were 256 and 128, respectively. *f* (activation function) was ReLU. We used batch normalization (Ioffe and Szegedy 2015) after each BICL in the ResBICL and each of fully connected layers. Also, we used dropout (Srivastava *et al.* 2014) with the drop rate of 0.25 to avoid overfitting. During the training process, the batch size was 128, the number of epochs was 50, and the optimizer we used was Adadelta (Zeiler 2012) with a weight decay of 1e−4. The learning rate began at 0.9 and a scheduler was used with the patience of 5 epochs. We implemented DeepMHCI by PyTorch (Paszke *et al.* 2019).

Although a large number of MHC-I-peptide binding prediction methods have been proposed in recent years, different methods tended to use different training sets. This often leads to biased comparisons and fails to demonstrate the real capability of models. In contrast, we conducted a 5-fold CV experiment where all methods were retrained on the same dataset, BD2017. Specifically, we compared DeepMHCI with the following state-of-the-art methods: NetMHCpan-3.0, DeepLigand-BA, DeepAttentionPan, and TransPHLA. Since the source code of NetMHCpan-3.0 is unavailable, we used its experimental results on BD2017 directly and downloaded the standalone version from the server for test. We trained DeepLigand-BA, DeepAttentionPan, and TransPHLA on BD2017 using the implementation provided by the authors. For a fair comparison, all methods used a bagging ensemble of 50 models.

Additionally, since two other state-of-the-art methods, ACME and DBTPred, were developed by the same lab, the latest method DBTpred was considered. DBTpred is a DBT-based method and is not designed in an end-to-end manner. Due to the difficulty of retraining DBTpred (https://github.com/fpy94/DBT/issues/1), we used the trained models provided by the authors directly. Furthermore, the training set of DBTpred is a subset of BD2017 with a random 5-fold split, which only contains HLA alleles (https://github.com/fpy94/DBT/tree/master/data_for_test). For a fair comparison, we also trained a variant of DeepMHCI on the training set of DBTpred with the same 5-fold split and used the same number of models, 5, for the ensemble.

### 3.3 Evaluation metrics

We used the default evaluation metrics in the original paper of each benchmark for performance comparison. Specifically, for the binary classification of binder and nonbinder, we used the area under the receiver operating characteristics curve (AUC) for each MHC-I molecule under each peptide length and reported the average AUC, respectively. To classify peptides into binders and nonbinders, all peptides with an IC50-binding value <500 nM (0.426 after transformation) were classified as binders. For BD2017, following the setting of NetMHCpan-3.0, only molecules with >20 data points and at least three binders were reported. Note that binding affinities were available for all data points in BD2017, the Pearson correlation coefficient (PCC) was used to examine the correlation between the predicted binding affinity and the true value.

As ID2022 is a collection of heterogeneous datasets from different labs and many of them don’t have binding affinities, spearman rank correlation coefficient (SRCC) instead of PCC was used (Trolle *et al.* 2015). In addition, a recent overall percent rank metric proposed for IEDB automated benchmark analysis (Trevizani *et al.* 2022) was used for evaluation. The overall value of *M* metrics for *D* datasets can be calculated as:

$$Ov(m_{1},\ldots ,m_{M})=\frac{1}{MD}\times \sum_{i=1}^{M}\sum_{j=1}^{D}PR(m_{i}(d_{j})),$$

where $PR(m_{i}(d_{j}))$ is the percent rank of metric *m_i_* on dataset *d_j_*, which can be calculated as:

$$PR(m_{i}(d_{j}))=(\frac{n_{l}-1}{N-1})\times 100,$$

where *n_l_* is the number of methods with an equal or lower performance and *N* is the number of methods. Specifically, we used *Ov*(AUC, SRCC) for evaluation over ID2022.

To evaluate the performance on epitope classification, following the setting of NetMHCpan (Jurtz *et al.* 2017), a Frank value was calculated for each entry. Specifically, denote the number of peptides with prediction scores higher than the positive peptide (epitope) as *n_p_*, and the number of peptides contained within the source protein as *N_all_*. The Frank value can be calculated as $n_{p}/N_{\mathrm{all}}$. Therefore, the lower the value of Frank, the better the result is.

### 3.4 Experimental results

We conducted the following four experiments to validate the predictive performance of DeepMHCI: (i) We examined the performance of DeepMHCI and the competing methods by 5-fold CV over BD2017. (ii) We performed performance comparisons of DeepMHCI and competing methods on the independent test set ID2022. (iii) We examined the performance of DeepMHCI and competing methods on an external dataset HPV2019 which contains experimentally validated HLA-peptide binding data. (iv) We examined the performance of DeepMHCI and the competing methods on an external dataset EP2017 to evaluate the ability of CD8+ epitope identification.

#### 3.4.1 Comparisons of DeepMHCI and competing methods under 5-fold cross-validation

Table 2 reports the average AUC and PCC over all MHC-I molecules of 5-fold CV over BD2017 by DeepMHCI, NetMHCpan-3.0, DeepLigand-BA, DeepAttentionPan, and TransPHLA. We can see that DeepMHCI outperformed all competing methods in both AUC and PCC at all lengths. Detailed results are shown in Supplementary Tables S3–S7. Specifically, DeepMHCI achieved the highest average PCC of 0.773 over 10-mer, which was followed by NetMHCpan-3.0 (0.761), TrasPHLA (0.760), DeepAttentionPan (0.743), and DeepLigand-BA (0.702). In addition, we found that the performance of DeepLigand-BA, DeepAttentionPan, and TransPHLA drop significantly on 11-mer and ≥12-mer, while the performance of DeepMHCI drops only slightly. For example, DeepMHCI outperformed the latest state-of-the-art method TransPHLA significantly, using a powerful Transformer-based network, on 11-mer and ≥12-mer. Specifically, in terms of PCC, the performance of DeepMHCI was 8.1% and 37.0% higher than those of TransPHLA at the length of 11 and ≥12, respectively, which reflects the advantage of DeepMHCI in handling non-9-mer peptides.

**Table 2.** Performance of DeepMHCI and competing methods on 5-CV dataset BD2017.

Method	8-mer		9-mer		10-mer		11-mer		≥ 12-mer	
	AUC	PCC	AUC	PCC	AUC	PCC	AUC	PCC	AUC	PCC
NetMHCpan-3.0^a^	0.896	0.727	0.896	0.726	0.892	0.761	0.885	0.712	0.832	0.643
DeepLigand-BA	0.858	0.639	0.865	0.679	0.864	0.702	0.817	0.548	0.734	0.415
DeepAttentionPan	0.878	0.676	0.895	0.726	0.882	0.743	0.857	0.645	0.771	0.503
TransPHLA	0.888	0.707	0.896	0.737	0.887	0.760	0.874	0.667	0.779	0.486
DeepMHCI	**0.904**	**0.743**	**0.904**	**0.752**	**0.897**	**0.773**	**0.895**	**0.721**	**0.845**	**0.666**

a Results of methods are taken from Nielsen and Andreatta (2016).

The best results are highlighted in bold.

#### 3.4.2 Comparisons of DeepMHCI and competing methods on independent test set

Table 3 reports *Ov*(AUC, SRCC), the average AUC and SRCC of each method on the independent test set, ID2022. ID2022 consists of 64 benchmarks covering 26 MHC molecules, with 51 datasets of 9-mer, 10 datasets of 10-mer, 2 datasets of 8-mer, and 1 dataset of 11-mer. Although most of them are datasets of 9-mer, DeepMHCI outperformed all competing methods on both average AUC and average SRCC over ID2022. Detailed results are shown in Supplementary Table S8. Specifically, DeepMHCI achieved the best average SRCC of 0.569, which was 6.2%, 18.5%, 3.1%, and 4.4% higher than NetMHCpan3.0 (0.536), DeepLigand-BA (0.480), DeepAttentionPan (0.552), and TransPHLA (0.545), respectively. In addition, for the overall metric *Ov*(AUC, SRCC), DeepMHCI achieved the highest value of 74.2, which was followed by DeepAttentionPan (67.1), TransPHLA (59.6), NetMHCpan-3.0 (58.8), and DeepLigand-BA (38.5).

**Table 3.** Performance of DeepMHCI and competing methods on ID2022.

Metric	NetMHCpan-3.0	DeepLigand-BA	DeepAttentionPan	TransPHLA	DeepMHCI
AUC	0.830	0.794	0.843	0.836	**0.846**
SRCC	0.536	0.480	0.552	0.545	**0.569**
*Ov*(AUC, SRCC)	58.8	38.5	67.1	59.6	**74.2**
*P*-value	$4.7\times 10^{-3}$	$7.7\times 10^{-5}$	$4.6\times 10^{-2}$	$2.7\times 10^{-2}$	
	(34/49)	(43/57)	(32/51)	(34/53)	

The best results are highlighted in bold.

Furthermore, considering the heterogeneity of 64 benchmark datasets, we use the statistical test to measure the significance of performance difference between DeepMHCI and other competing methods with respect to SRCC. Similar to Nielsen *et al.* (2007b), for comparing two predictors, we used one-tailed binomial test to measure the performance differences (excluding ties), where *P*-value <.05 is considered to being statistically significant. As shown in the lower part of Table 3, DeepMHCI outperformed all other competing methods, being statistically significant. For example, DeepMHCI outperformed NetMHCpan-3.0 on 34 of the 49 datasets (excluding 15 ties), TransPHLA on 34 of the 53 datasets (excluding 11 ties), and DeepAttentionPan on 32 of the 51 datasets (excluding 13 ties). All of them are statistically significant (one-tailed binomial test, with *P*-value = $4.7\times 10^{-3},2.7\times 10^{-2}$, and $1.5\times 10^{-4}$, respectively). DeepMHCI also outperformed DBTPred significantly by using five models for ensemble on ID2022. Detailed results are shown in Supplementary Tables S2 and S9. All these results highlight the superiority and robustness of DeepMHCI.

#### 3.4.3 Comparisons of DeepMHCI and competing methods on HPV vaccine identification

**Figure 2.**
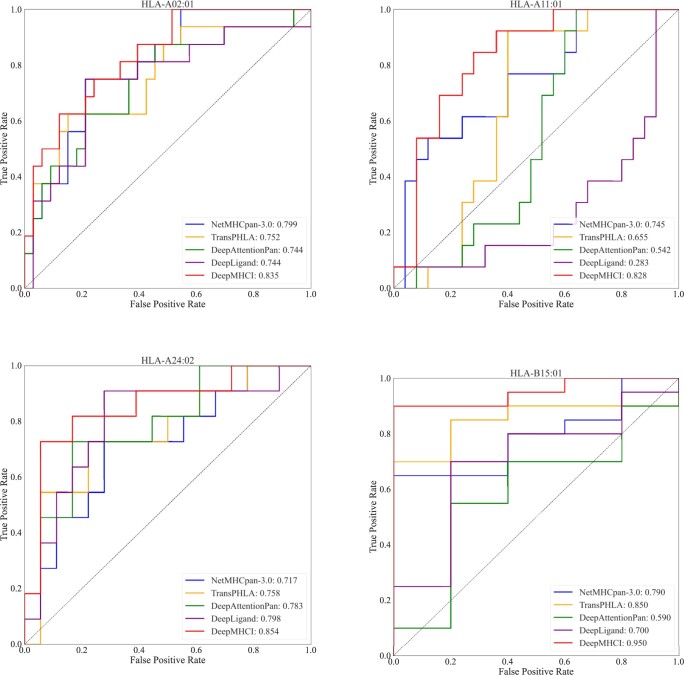
ROC curves by MHC-I molecules in HPV2019 of 11-mer.

Table 4 reports the average AUC over all MHC-I molecules of HPV2019 by different methods. The upper part shows the performance of DeepMHCI, NetMHCpan-3.0, DeepLigand-BA, DeepAttentionPan, and TransPHLA with the ensemble of 50 models, while the lower part shows the performance of DeepMHCI and DBTpred with the ensemble of 5 models. Detailed results are shown in Supplementary Tables S10–S13. We can see that DeepMHCI achieved the best results on 10-mer and 11-mer, and ranked second on 8-mer and 9-mer. For example, in the upper part, DeepMHCI achieved the highest AUC in both 10-mer and 11-mer, and the second best in both 8-mer and 9-mer. In particular for longer peptides with a length of 11-mer, which need more complex anchor residue awareness, DeepMHCI achieved the best average AUC of 0.751, which were 9.0% and 13.6% higher than those achieved by NetMHCpan-3.0 (0.689) and TransPHLA (0.661), the second and third best methods, respectively. Figure 2 plots the AUC curves of all molecules (excluding one molecule with an AUC of <0.5 for all methods) with 11-mer, where DeepMHCI achieved the best performance on all four molecules. The results for the remaining molecule are shown in Supplementary Fig. S1. The overall experimental results again verify that, by incorporating biological knowledge into the model architecture for anchor residue position awareness, DeepMHCI achieves a substantial improvement in prediction accuracy at non-9-mer peptides.

**Table 4.** Performance of DeepMHCI and competing methods on HPV2019.

Method	8mer-AUC	9mer-AUC	10mer-AUC	11mer-AUC
NetMHCpan-3.0	**0.869**	0.892	0.840	0.689
DeepLigand-BA	0.587	0.854	0.838	0.592
DeepAttentionPan	0.677	**0.905**	0.864	0.598
TransPHLA	0.723	0.884	0.876	0.661
DeepMHCI	0.863	0.895	**0.877**	**0.751**
DBTpred^a^	**0.891**	0.884	0.822	0.640
DeepMHCI^a^	0.858	**0.893**	**0.854**	**0.701**

a Results of methods were only used five models to the ensemble.

The best results are highlighted in bold.

#### 3.4.4 Comparisons of DeepMHCI and competing methods on epitope classification

To further evaluate the performance on CD8+ epitopes identification, we specifically compared the performance between DeepMHCI with the three methods that have been tested with better performance in the above experiments, TransPHLA, NetMHCpan-3.0, and DeepAttentionPan. Table 5 reports the average AUC and Frank over all MHC-I molecules of EP2017 by all these methods. Detailed results are shown in Supplementary Tables S14–S20. We can see that DeepMHCI achieved the highest AUC of 0.980, and the lowest (best) Frank of 1.98%. Furthermore, we examined the performance of different methods over epitopes with different lengths (8, 9, 10, 11, 12, and 13). DeepMHCI achieved the best Frank over 9-mer, 11-mer, 12-mer, and 13-mer, and the second-best Frank over 8-mer and 10-mer. The improvement is especially significant in the case of longer peptides of length 11, 12, and 13, where more complex perceptions of anchor residues are needed. For example, DeepMHCI achieved the lowest Frank of 1.57% over 12-mer, which was followed by NetMHCpan-3.0 (2.03%), TransPHLA (5.26%), and DeepAttentionPan (9.96%).

**Table 5.** Performance of DeepMHCI and competing methods on EP2017.

Method	AUC	Frank	Frank_8_	Frank_9_	Frank_10_	Frank_11_	Frank_12_	Frank_13_
DeepAttentionPan	0.975	2.54%	2.08%	2.15%	2.76%	2.68%	9.96%	2.30%
TransPHLA	0.978	2.22%	1.52%	2.17%	2.39%	2.35%	5.26%	0.91%
NetMHCpan-3.0	0.979	2.05%	**1.15%**	2.06%	**2.03%**	2.34%	2.03%	0.21%
DeepMHCI	**0.980**	**1.98%**	1.36%	**1.95%**	2.35%	**2.17%**	**1.57%**	**0.06%**

The best results are highlighted in bold.

## 4 Result analysis

### 4.1 Binding motifs analysis

**Figure 3.**
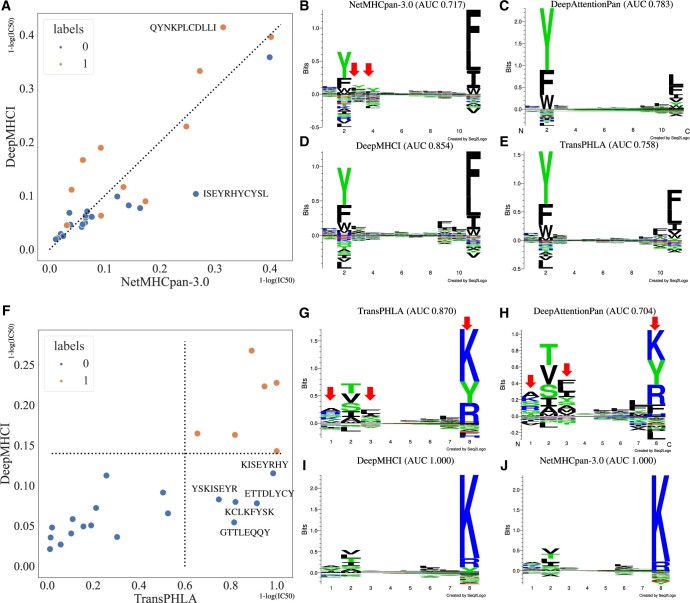
Motifs comparison between DeepAttentionPan, TransPHLA, NetMHCpan-3.0, and DeepMHCI. (A) The scatter distribution of predicted values of DeepMHCI and NetMHCpan-3.0 under the 11-mer of HLA-A24:02 in the HPV2019. (B–E) The motif of HLA-A24:02 under 11-mer by NetMHCpan-3.0, DeepAttentionPan, DeepMHCI, and TransPHLA, respectively. (F) The scatter distribution of predicted values of DeepMHCI and TransPHLA under the 8-mer of HLA-A11:01 in the HPV2019. (G–J) The motif of HLA-A11:01 under 8-mer by TransPHLA, DeepAttentionPan, DeepMHCI, and NetMHCpan-3.0, respectively.

To understand the superiority of DeepMHCI, we visualized the binding motifs of MHC-I molecules by using sequence logos (Schneider and Stephens 1990) and compared the motifs generated by DeepMHCI with those of DeepAttentionPan, TransPHLA, and NetMHCpan-3.0. We first computed the binding scores of 100 000 random peptides from SwissProt (Boeckmann *et al.* 2003) at a specific length and then selected the top 1% predicted binders to draw sequence logos (with default settings). In each sequence logo (Fig. 3B–E and G–J), the *x*-axis shows the alignment position, where at each position of a motif, the total height [of letters (amino acids)] describes the amount of information content (also importance), and the height of each letter shows the frequency of the corresponding observed amino acids in the position. We focus on the molecules that DeepMHCI consistently performs well under non-9-mer on the external dataset HPV2019, but DeepAttentionPan, TransPHLA and NetMHCpan-3.0 perform poorly. In particular, we selected HLA-A24:02 and HLA-A11:01 for illustration. All motifs of length 8–11 by different methods are also shown in Supplementary Fig. S2. More comparative analysis of generated binding motifs of other MHC-I molecules can be found in Supplementary materials.

Figure 3A plots the scatter distribution of predicted values of both NetMHCpan-3.0 (*x*-axis) and DeepMHCI (*y*-axis) under 11-mer of HLA-A24:02 for HPV2019. We can see that DeepMHCI predicts higher values than NetMHCpan-3.0 for positive samples. Two outliers are marked, QYNKPLCDLLI predicted with higher score by DeepMHCI and ISEYRHYCYSL predicted as a false positive sample by NetMHCpan-3.0. Comparing the motifs (Fig. 3B and D) of the two methods, we find that DeepMHCI has a more obvious amino acid preference for L and L at positions 9 and 10. This can explain why DeepMHCI favors QYNKPLCDLLI. For the second outlier, the higher prediction value given by NetMHCpan-3.0 is due to its strategy in handling peptides over 9-mer. NetMHCpan-3.0 first converts the target peptide to a pseudo peptide of 9-mer, allowing consecutive deletions, then predicts binding affinity based on the 9-mer subsequence. The subsequence IYRHYCYSL obtained by deleting the second and third amino acids in the ISEYRHYCYSL exactly matches the binding motif of NetMHCpan-3.0, where the amino acid Y appears at the first anchor position (the second position). This oversimplified handling of peptides over 9-mer is directly reflected in the binding motif (Fig. 3B); the amino acid Y preference at positions 3 and 4 is the duplication of the preference at position 2 (the real anchor position), which does not exist. This phenomenon may account for the fact that NetMHCpan-3.0 achieved poor performances under 11-mer of HLA-A24:02 (AUC 0.717), which are much lower than those of DeepMHCI (AUC 0.854). In addition, the motifs of DeepAttentionPan (Fig. 3C) and TransPHLA (Fig. 3E) differ from DeepMHCI mainly in the positions 9-11, which may account for their better performance than NetMHCpan-3.0.

Figure 3F also plots the scatter distribution of predicted values of both TransPHLA (*x*-axis) and DeepMHCI (*y*-axis) under 8-mer of HLA-A11:01 for HPV2019. We can see that DeepMHCI could completely distinguish positive from negative examples, with all positive samples appearing in the upper right area. However, TransPHLA produces a large number of false positive samples in the lower right area. Comparing these false positive peptides against the motifs (Fig. 3G and I) yields the following findings. (i) Amino acid Y frequently occurs at position 8 of false positive samples, consistent with the motif of TransPHLA having a higher preference for amino acid Y at position 8. (ii) Amino acid K appears at position 1 of some false positive samples and the motif of TransPHLA also shows a consistent preference for K. In contrast, the motif of DeepMHCI does not have this preference. (iii) Amino acids T and L appear at position 3 of some false positive samples, which is consistent with the motif of TransPHLA. In contrast, the third position of 8-mer is not considered as an anchor position by DeepMHCI. Furthermore, we find that the motifs of TransPHLA (Fig. 3G) and DeepAttentionPan (Fig. 3H) are similar; both have similar amino acid preferences at position 1, 2, 3, and 8. While the motifs of DeepMHCI (Fig. 3I) and NetMHCpan-3.0 (Fig. 3J) are almost identical, with similar amino acid preferences at position 1, 2, 6, and 8. These contrasting motifs may account for the differences in performance that both DeepMHCI and NetMHCpan-3.0 achieved 1.0 of AUC, which are much higher than those of TransPHLA (AUC 0.870) and DeepAttentionPan (AUC 0.704).

We further conducted a comparative analysis of the generated motifs of HLA supertypes (Sette and Sidney 1999, Lund *et al.* 2004, Sidney *et al.* 2008, Nguyen *et al.* 2021) by DeepMHCI. Our results confirmed that the MHC-I molecules within the same HLA supertype share a majority of anchor positions with similar amino acid preferences. In contrast, motifs displayed by different HLA supertypes exhibit significant differences. Detailed results are shown in Supplementary materials.

### 4.2 Ablation study

We first examined DeepMHCI with different combination of modules. Table 6 reports the average performance over each length of 10 times 5-fold CV by each combination over BD2017. We have two findings: (i) both position-wise gated layer and residual BICL can improve performance and (ii) applying both modules simultaneously, DeepMHCI achieved the best performance. These results confirm that the high performance of DeepMHCI is attributed to the informative control of anchor positions by the gated layer and robustness of ResBICL.

**Table 6.** Performance of DeepMHCI with different module combinations over BD2017.

Method	AUC	PCC
BICL	0.884	0.716
+ Gated Layer	0.885	0.725
+ ResBICL	0.887	0.727
DeepMHCI	**0.889**	**0.731**

The best results are highlighted in bold.

We also examined the performance of DeepMHCI using individual kernel sizes and elaborated the reasons for using kernel size with *k* = 9, 11, 13. Since most MHC-I molecules prefer to bind 9-mer long peptides, we chose *k* = 9 as the most basic convolution kernel size (with the largest number). Considering the amino acid sequence beyond the 9-mer part, we naturally tried to use larger convolution kernel sizes for coverage. Specifically, we used *k* = 11 and 13, and combined with padding operations to maintain the output shape of BICL. At the same time, considering that the vast majority of binding peptide sequences are no longer than 13, we did not use larger convolution sizes. We then selected models with kernel sizes *k *=* *9, 11, 13, which was named DeepMHCI_*k*_ for comparison with DeepMHCI using all kernel size. The results are shown in Supplementary Table S21. We found that DeepMHCI with mixing kernels of different sizes achieved the best performance.

In addition, we explored the effect of different amino acid embedding options on the performance. We found that a learned embedding was the optimal choice. The results are shown in Supplementary Table S22.

## 5 Conclusion and discussion

In this work, we propose a new deep learning model, DeepMHCI, that demonstrates more accurate predictions of the binding affinities between peptides of general length (especially non-9-mers) and MHC-I molecules. In terms of model design, this is made possible by two novel features, the position-wise gated layer and the residual binding interaction convolution layer. Given these properties, adaptive awareness of the anchor position forms the basis of the model, which exhibits more accurate motifs under different lengths to understand the peptide-MHC binding pattern.

Through extensive experimental analysis, we found that DeepMHCI outperformed all five state-of-the-art competing methods, especially on non-9-mer peptides that have not been well studied for a long time. DeepMHCI can be naturally extended to epitope prediction and we expect it can provide effective assistance in discovering personalized cancer neoantigen in clinical diagnosis(Ott *et al.* 2017, Reynisson *et al.* 2020).

## Supplementary Material

btad551_Supplementary_Data

## Data Availability

The dataset BD2017 is available at https://services.healthtech.dtu.dk/suppl/immunology/NetMHCpan-4.0/. The dataset ID2021 is available at http://tools.iedb.org/auto_bench/mhci/weekly/. The dataset EP2017 is available at https://services.healthtech.dtu.dk/service.php?NetMHCpan-4.1. The dataset HPV2019 could be downloaded from the supplementary information at (Bonsack *et al.* 2019).

## References

[btad551-B1] Andreatta M , Nielsen M. Gapped sequence alignment using artificial neural networks: application to the MHC class I system. Bioinformatics 2016;32:511–7. 26515819 10.1093/bioinformatics/btv639 PMC6402319

[btad551-B2] Anjanappa R , Garcia-Alai M, Kopicki J-D et al Structures of peptide-free and partially loaded MHC class I molecules reveal mechanisms of peptide selection. Nat Commun 2020;11:1314. 32161266 10.1038/s41467-020-14862-4 PMC7066147

[btad551-B3] Boeckmann B , Bairoch A, Apweiler R et al The SWISS-PROT protein knowledgebase and its supplement TrEMBL in 2003. Nucleic Acids Res 2003;31:365–70. 12520024 10.1093/nar/gkg095 PMC165542

[btad551-B4] Bonsack M , Hoppe S, Winter J et al Performance evaluation of MHC class-I binding prediction tools based on an experimentally validated MHC-peptide binding data set. Cancer Immunol Res 2019;7:719–36. 30902818 10.1158/2326-6066.CIR-18-0584

[btad551-B5] Chu Y , Zhang Y, Wang Q et al A transformer-based model to predict peptide-HLA class I binding and optimize mutated peptides for vaccine design. Nat Mach Intell 2022;4:300–11.

[btad551-B6] Dauphin YN , Fan A, Auli M et al Language modeling with gated convolutional networks. In: *International Conference on Machine Learning, International Convention Centre, Sydney, Australia, 6–11 August 2017*, 2017, 933–41. PMLR.

[btad551-B7] Feng P , Zeng J, Ma J. Predicting MHC-peptide binding affinity by differential boundary tree. Bioinformatics 2021;37:i254–61. 34252932 10.1093/bioinformatics/btab312 PMC8275335

[btad551-B8] Hu Y , Wang Z, Hu H et al ACME: pan-specific peptide-MHC class I binding prediction through attention-based deep neural networks. Bioinformatics 2019;35:4946–54. 31120490 10.1093/bioinformatics/btz427

[btad551-B9] Ioffe S , Szegedy C. Batch normalization: accelerating deep network training by reducing internal covariate shift. In: *International Conference on Machine Learning, Lille, France, 7–9 July 2015*, 2015, 448–56. PMLR.

[btad551-B10] Janeway CA , Travers P, Walport M et al 2005. Immunobiology: The Immune System in Health and Disease. 6th edn. New York: Garland Science Publishing.

[btad551-B11] Jin J , Liu Z, Nasiri A et al Deep learning pan-specific model for interpretable MHC-I peptide binding prediction with improved attention mechanism. Proteins Struct FunctBioinform 2021;89:866–83. 10.1002/prot.26065 33594723

[btad551-B12] Jurtz VI , Paul S, Andreatta M et al NetMHCpan-4.0: improved peptide-MHC class I interaction predictions integrating eluted ligand and peptide binding affinity data. J Immunol 2017;199:3360–8. 28978689 10.4049/jimmunol.1700893 PMC5679736

[btad551-B13] Lund O , Nielsen M, Kesmir C et al Definition of supertypes for HLA molecules using clustering of specificity matrices. Immunogenetics 2004;55:797–810. 14963618 10.1007/s00251-004-0647-4

[btad551-B14] Mei S , Li F, Xiang D et al Anthem: a user customised tool for fast and accurate prediction of binding between peptides and HLA class I molecules. Brief Bioinform 2021;22:bbaa415. 33454737 10.1093/bib/bbaa415

[btad551-B15] Nguyen AT , Szeto C, Gras S. The pockets guide to HLA class I molecules. Biochem Soc Trans 2021;49:2319–31. 34581761 10.1042/BST20210410 PMC8589423

[btad551-B16] Nielsen M , Andreatta M. NetMHCpan-3.0; improved prediction of binding to MHC class I molecules integrating information from multiple receptor and peptide length datasets. Genome Med 2016;8:33–9. 27029192 10.1186/s13073-016-0288-x PMC4812631

[btad551-B17] Nielsen M , Lundegaard C, Blicher T et al NetMHCpan, a method for quantitative predictions of peptide binding to any HLA-A and-B locus protein of known sequence. PLoS One2007a;2:e796. 17726526 10.1371/journal.pone.0000796 PMC1949492

[btad551-B18] Nielsen M , Lundegaard C, Lund O. Prediction of MHC class II binding affinity using SMM-align, a novel stabilization matrix alignment method. BMC Bioinform. 2007b;8:238–12. 10.1186/1471-2105-8-238 PMC1939856 17608956

[btad551-B19] O’Donnell TJ , Rubinsteyn A, Bonsack M et al MHCflurry: open-source class I MHC binding affinity prediction. Cell Syst 2018;7:129–32.e4. 29960884 10.1016/j.cels.2018.05.014

[btad551-B20] Ott PA , Hu Z, Keskin DB et al An immunogenic personal neoantigen vaccine for patients with melanoma. Nature 2017;547:217–21. 28678778 10.1038/nature22991 PMC5577644

[btad551-B21] Paszke A , Gross S, Massa F et al Pytorch: an imperative style, high-performance deep learning library. NeurIPS 2019;32:8024–35.

[btad551-B22] Reynisson B , Alvarez B, Paul S et al NetMHCpan-4.1 and NetMHCIIpan-4.0: improved predictions of MHC antigen presentation by concurrent motif deconvolution and integration of MS MHC eluted ligand data. Nucleic Acids Res 2020;48:W449–54. 32406916 10.1093/nar/gkaa379 PMC7319546

[btad551-B23] Schneider TD , Stephens RM. Sequence logos: a new way to display consensus sequences. Nucleic Acids Res 1990;18:6097–100. 2172928 10.1093/nar/18.20.6097 PMC332411

[btad551-B24] Sette A , Sidney J. Nine major HLA class I supertypes account for the vast preponderance of HLA-A and-B polymorphism. Immunogenetics 1999;50:201–12. 10602880 10.1007/s002510050594

[btad551-B25] Sidney J , Peters B, Frahm N et al HLA class I supertypes: a revised and updated classification. BMC Immunol 2008;9:1–15. 18211710 10.1186/1471-2172-9-1 PMC2245908

[btad551-B26] Srivastava N , Hinton G, Krizhevsky A et al Dropout: a simple way to prevent neural networks from overfitting. J Mach Learn Res 2014;15:1929–58.

[btad551-B27] Trevizani R , Yan Z, Greenbaum JA et al A comprehensive analysis of the IEDB MHC class-I automated benchmark. Brief Bioinform 2022;23:bbac259. 35794711 10.1093/bib/bbac259 PMC9618166

[btad551-B28] Trolle T , Metushi IG, Greenbaum JA et al Automated benchmarking of peptide-MHC class I binding predictions. Bioinformatics 2015;31:2174–81. 25717196 10.1093/bioinformatics/btv123 PMC4481849

[btad551-B29] Vita R , Mahajan S, Overton JA et al The immune epitope database (IEDB): 2018 update. Nucleic Acids Res 2019;47:D339–43. 30357391 10.1093/nar/gky1006 PMC6324067

[btad551-B30] You R , Qu W, Mamitsuka H et al DeepMHCII: a novel binding core-aware deep interaction model for accurate MHC-II peptide binding affinity prediction. Bioinformatics 2022;38:i220–8. 35758790 10.1093/bioinformatics/btac225 PMC9235502

[btad551-B31] Zeiler MD. Adadelta: an adaptive learning rate method. arXiv, arXiv:1212.5701, 2012, Vol. 32, 8024–35, preprint: not peer reviewed.

[btad551-B32] Zeng H , Gifford DK. DeepLigand: accurate prediction of MHC class I ligands using peptide embedding. Bioinformatics 2019;35:i278–83. 31510651 10.1093/bioinformatics/btz330 PMC6612839

[btad551-B33] Zhang L , Udaka K, Mamitsuka H et al Toward more accurate pan-specific MHC-peptide binding prediction: a review of current methods and tools. Brief Bioinform 2012;13:350–64. 21949215 10.1093/bib/bbr060

