# Supplementary material for: DeepMHCI: an anchor position-aware deep interaction model for accurate MHC-I peptide binding affinity prediction
Source: Bioinformatics. 2023 Sep 5;39(9):btad551. doi: 10.1093/bioinformatics/btad551 (PMC10516514; doi:10.1093/bioinformatics/btad551)
Supplement: btad551_Supplementary_Data [file btad551_supplementary_data.pdf]

Supplementary Materials for

DeepMHCI: An anchor position-aware deep interaction model for accurate MHC-I peptide binding affinity prediction

Wei Qu[1], Ronghui You[1], Hiroshi Mamitsuka[2], and Shanfeng Zhu *[1]

[1]Institute of Science and Technology for Brain-Inspired Intelligence, Fudan University
[2]Bioinformatics Center, Institute for Chemical Research, Kyoto University

1 Data Analysis

We have evaluated the redundancy of the 5-fold CV split of BD2017 by comparing it with a random split. Outer-P is the probability that two peptides in different folds have a common 8-mer subsequence, while Inner-P is the probability that two peptides in the same fold have a common 8-mer subsequence. As shown in Table S1, the original strict split results in low redundancy, since Outer-P is only around 0.7% of Inner-P while Outer-P and Inner-P have the same value for a random split.

Table S1: Data redundancy of BD2017

Split	Outer-P	Inner-P	Inner-P / Outer-P
Strict	6.68e-6	8.91e-4	133.4
Random	1.84e-4	1.84e-4	1

2 Experimental Results

2.1 Detailed Results of Five Fold Cross-Validation over BD2017

Table S3 S4 S5 S6 S7 show the detailed five-fold cross-validation perfomance of DeepMHCI and competing methods for all MHC-I molecules under 8mer, 9mer, 10mer, 11mer and ≥12mer, respectively. DeepMHCI outperformed all competing methods in both AUC and PCC under all lengths.

2.2 Detailed Results over Independent Test Set ID2022

Table S8 shows the detailed results of DeepMHCI and competing methods over each benchmark of Independent test set ID2022. DeepMHCI achieved the highest averaged SRCC (0.569) and AUC (0.846), respectively.

We also compared with DBTpred using 5 models for ensemble over ID2022. Table S2 reports the average AUC and SRCC and Ov(AUC, SRCC) of DBTpred and DeepMHCI on the ID2022. DeepMHCI achieved better average SRCC of 0.563, which was 23.5% higher than DBTpred (0.456). Detailed results are shown in the Table S9.

*Corresponding author: zhusf@fudan.edu.cn

Table S2: Performance of DeepMHCI† and DBTpred† on ID2022. Both methods were only used 5 models to the ensemble.

Metric	DBTpred†	DeepMHCI†
AUC	0.775	**0.843**
SRCC	0.456	**0.563**
Ov(AUC, SRCC)	40.6	**75.8**
p-value	1.92×10^{-3} (38/54)	

2.3 Detailed Results of HPV Vaccine Identification over HPV2019

Table S10 S11 S12 S13 show the detailed results of DeepMHCI and competing methods over HPV2019 for all MHC-I molecules under 8mer, 9mer, 10mer and 11mer, respectively. DeepMHCI achieved almost the best results at all lengths. Specifically, DeepMHCI achieved the best mean AUC under 10mer and 11mer and the second best mean AUC under 9mer. Figure S1 plots ROC curves of the remaining MHC-I molecule on the HPV2019 dataset under 11-mer for DeepMHCI and comparison methods.

2.4 Detailed Results of Epitope Classification over EP2017

Table S14 S15 S16 S17 S18 S19 S20 show the detailed results of DeepMHCI and competing methods over EP2017 for all MHC-I molecules under all lengths, 8mer, 9mer, 10mer, 11mer, 12mer and 13mer, respectively. DeepMHCI performed much better in longer epitopes classification.

2.5 Detailed motifs

Figure S2 shows the motifs of various lengths of molecules for which DeepMHCI and competing methods have significantly different performance over HPV2019. Since the majority of MHC-I molecules are biased to bind 9-mer-long peptides, the binding motif of 9-mer can be considered as a reference. As can be seen in Figure S2, all four methods found common anchor positions of 9-mer binding peptides for different MHC-I molecules. However, there are significant differences among these methods for non-9-mer motifs. We summarized two main phenomena that may account for the low performance of DeepAttentionPan, TransPHLA and NetMHCpan-3.0 on these two molecules.

1) Wrong anchor positions discovery. In the motif of the HLA-A11:01 under 8-mer, both NetMHCpan-3.0 and DeepMHCI discovered consistent anchor positions at sites 1, 2, 6 and 8, which also showed consistent amino acid types. However, the anchor positions identified by TransPHLA and DeepAttentionPan were sites 1, 2, 3 and 8, where shows kind of different amino acid preference. These differences can explain the experimental results in HPV2019 under 8mer: both NetMHCpan-3.0 and DeepMHCI achieved extremely high performance (AUC 1.0 for both), much higher than those of TransPHLA (AUC 0.870) and DeepAttentionPan (AUC 0.704).

2) Shifted motif. In the motif of HLA-A24:02 under 11-mer, the amino acid preferences of NetMHCpan-3.0 at positions 3 and 4 are highly consistent with that of the second site, which is an anchor position of 9-mer. The shifted motif comes from the collate function for non-9-mer peptides of NetMHCpan-3.0, which generates pseudo 9-mer peptides by deleting two amino acids from 11-mer peptides.

We further selected three MHC-I molecules, HLA-A01:01, HLA-A29:02, and H-2-Db, for illustration, based on DeepMHCI's good performance under non-9-mer on the 5-fold cross-validation. Figure S3 presents the motifs of different methods on HLA-A01:01 under the 10-mer condition. It is evident that NetMHCpan-3.0 has replicated the phenomenon of anchor site duplication. Specifically, the amino acid preference for D at positions 4 duplicates the preference at position 3 (the actual anchor position), which should not occur. This phenomenon might explain why NetMHCpan-3.0 achieved the lowest performances under 10-mer of HLA-A01:01 (AUC 0.910 and PCC 0.762), competing with those of TransPHLA (AUC 0.914 and PCC 0.785) and DeepMHCI (AUC 0.930 and PCC 0.804). Figure S4 illustrates the motifs on HLA-A29:02 under the 10-mer condition. Notably, DeepMHCI shows an additional amino acid preference for S, whereas other

methods exhibit the same amino acid preferences for F, L, V, Y, T, I, and M at position 2. Similarly, only DeepMHCI displays an additional amino acid preference for M at position 10. Additionally, only TransPHLA exhibits an amino acid preference for Y at position 9, which is not considered an anchor position by DeepMHCI and NetMHCpan-3.0. These observed differences may account for the results where DeepMHCI achieved the best performances under the 10-mer condition for HLA-A29:02 (AUC 0.881 and PCC 0.766), competing with those of NetMHCpan-3.0 (AUC 0.863 and PCC 0.735) and TransPHLA (AUC 0.862 and PCC 0.735). Figure S5 presents the motifs of different methods on H-2-Db under the 11-mer condition. Notably, TransPHLA and DeepAttentionPan do not exhibit a significant preference for amino acids at position 7, while DeepMHCI and NetMHCpan-3.0 show a preference for amino acids N and D at this position. Additionally, only NetMHCpan-3.0 displays a significant preference for amino acid N at position 6, which is not considered an anchor point by the other methods. Moreover, only DeepAttentionPan shows a preference for amino acid D at position 5, which is opposite to the preferences observed in other methods. These differences may explain the results, with DeepMHCI achieving the best performance (AUC 0.885 and PCC 0.595), followed by TransPHLA (AUC 0.847 and PCC 0.576), NetMHCpan-3.0 (AUC 0.847 and PCC 0.556), and finally DeepAttentionPan (AUC 0.826 and PCC 0.556).

2.6 Motifs of HLA supertypes

We further conducted a comparative analysis of the generated motifs of HLA supertypes by DeepMHCI. We selected different HLA supertypes [4, 1, 5, 2] to demonstrate this phenomenon: 1) the binding motifs of HLA molecules generated by DeepMHCI in the same supertype are very similar; 2) the motifs generated between different supertypes are quite different. In particular, we randomly selected five HLA molecules from HLA supertype A2 and B58 for illustration, respectively. Figure S6 illustrates the motifs generated by DeepMHCI. It is evident that the major anchor points are shared within the same HLA supertype: for the five molecules in A2, there is a preference for amino acids L, M, and I at the second position, and a preference for amino acids V, L, I, and A at the ninth position; for the five molecules in B2, there is a preference for amino acids S, A, and T at the second position and W, F, I, and L at the ninth position. Moreover, when comparing the two supertypes A2 and B58, significant differences can be observed in the generated motifs displayed by different supertypes.

2.7 Ablation Study

We also examined the performance of DeepMHCI using individual kernel sizes. We selected models with kernel sizes $k = 9, 11, 13$, which was named DeepMHCI$_k$ for comparison with DeepMHCI using all kernel size. Table S21 reports the average performance over each length of 10 times 5-fold CV by each model over BD2017. We found that DeepMHCI achieved the best performance, which suggests that mixing kernels of different sizes to cover and process peptides contributes to model robustness.

In addition, We explored the effect of different amino acid embedding options on the performance. TableS22 reports the average performance over each length of one time 5-fold CV by each option. Learnable embedding was much better than the BLOSUM50 matrix. Moreover, ESM-1 [3] did not achieve the expected high performance. This may be due to the lack of pMHC-related interaction information in the ESM-1 task, and the high-dimensional embedding information has become a bottleneck for training DeepMHCI.

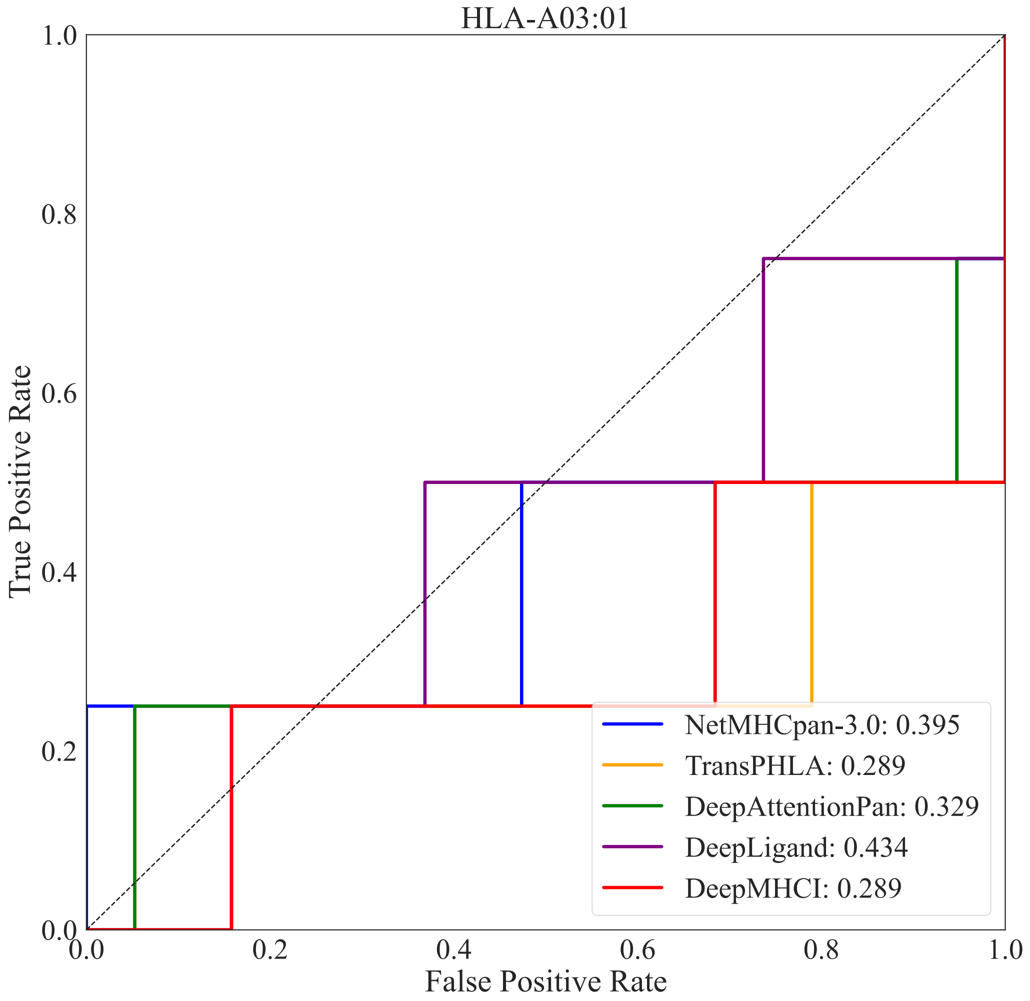

Figure S1: ROC curves by MHC-I molecules in HPV2019 of 11-mer.

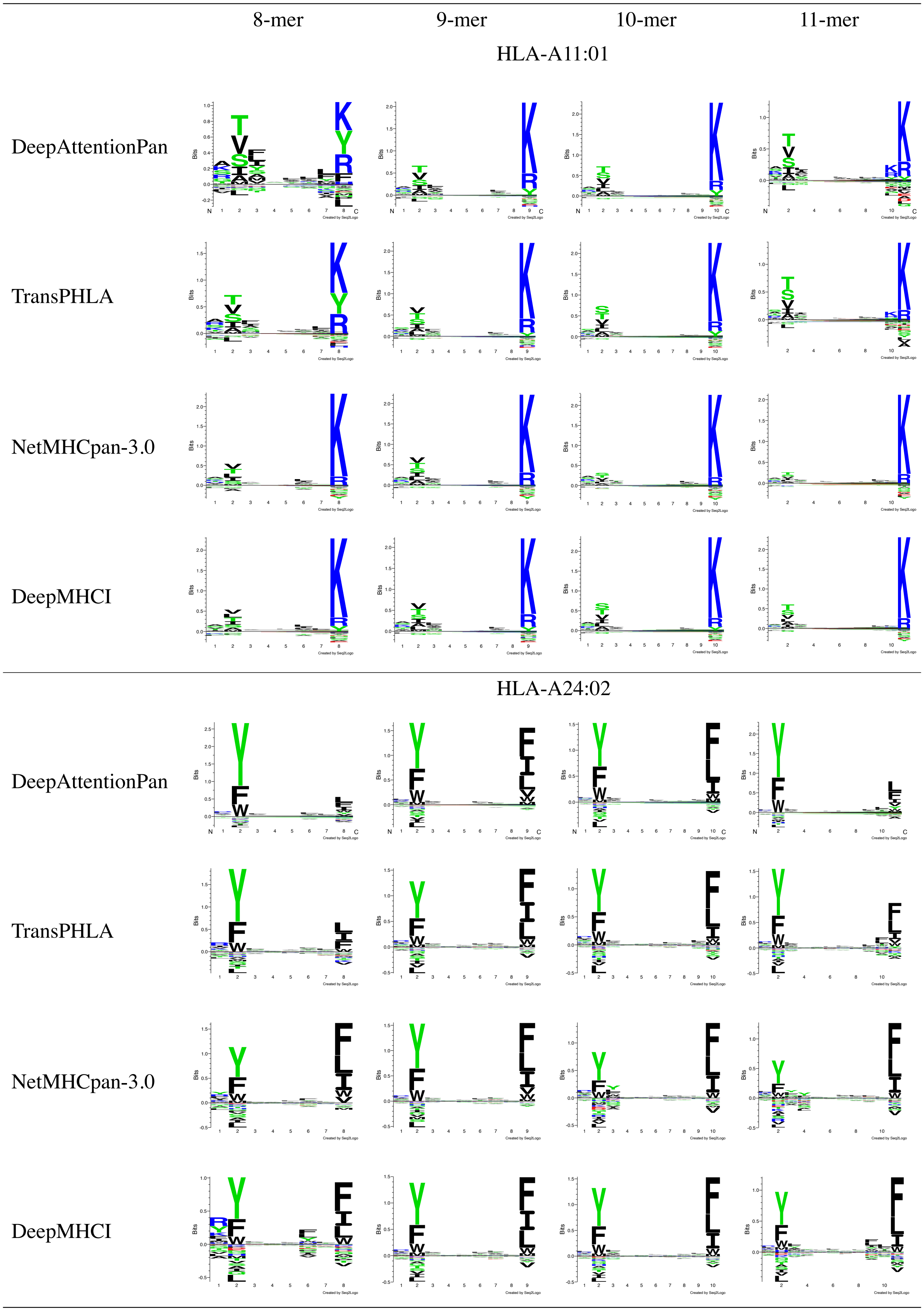

Figure S2: Motifs of DeepAttentionPan, TransPHLA, NetMHCpan-3.0 and DeepMHCI.

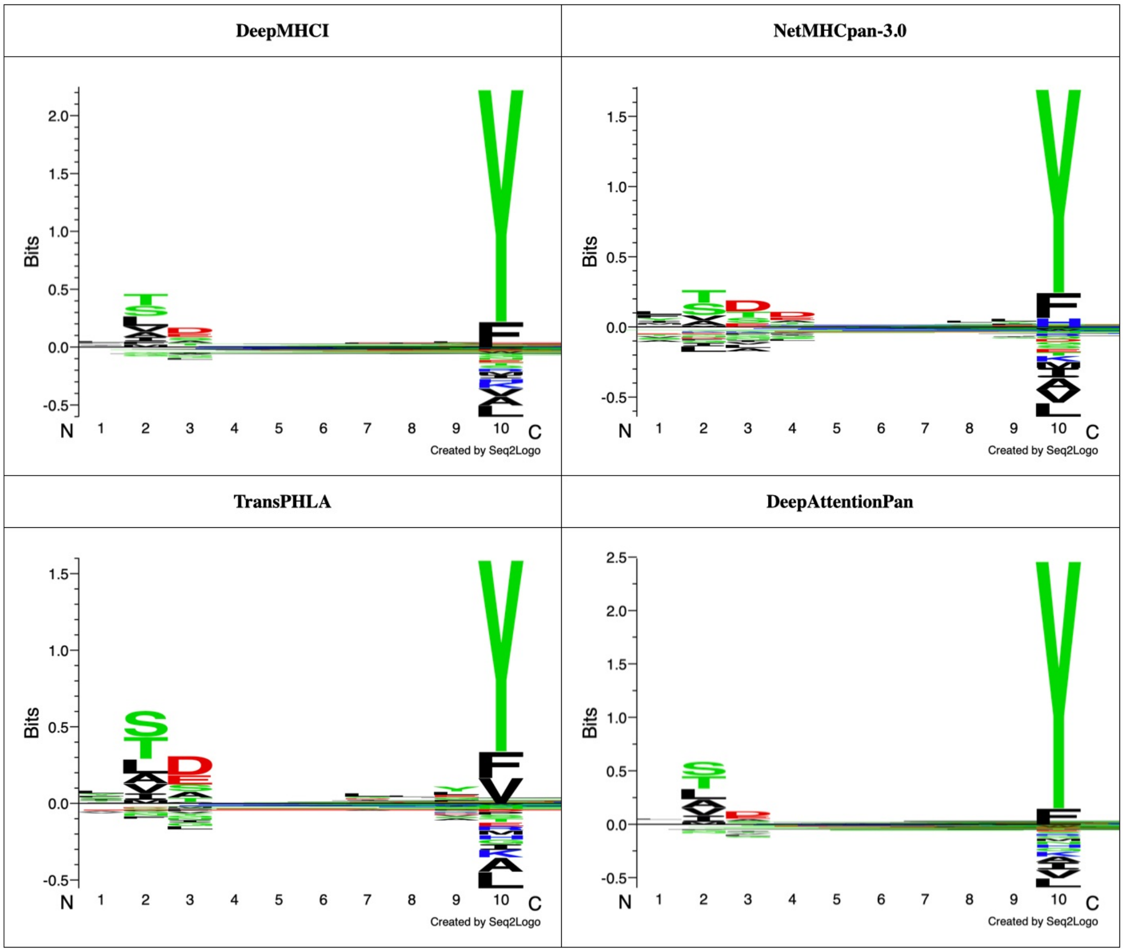

Figure S3: Motifs of DeepMHCI, NetMHCpanpan-3.0, TransPHLA and DeepAttentionPan on HLA-A01:01 under 10-mer.

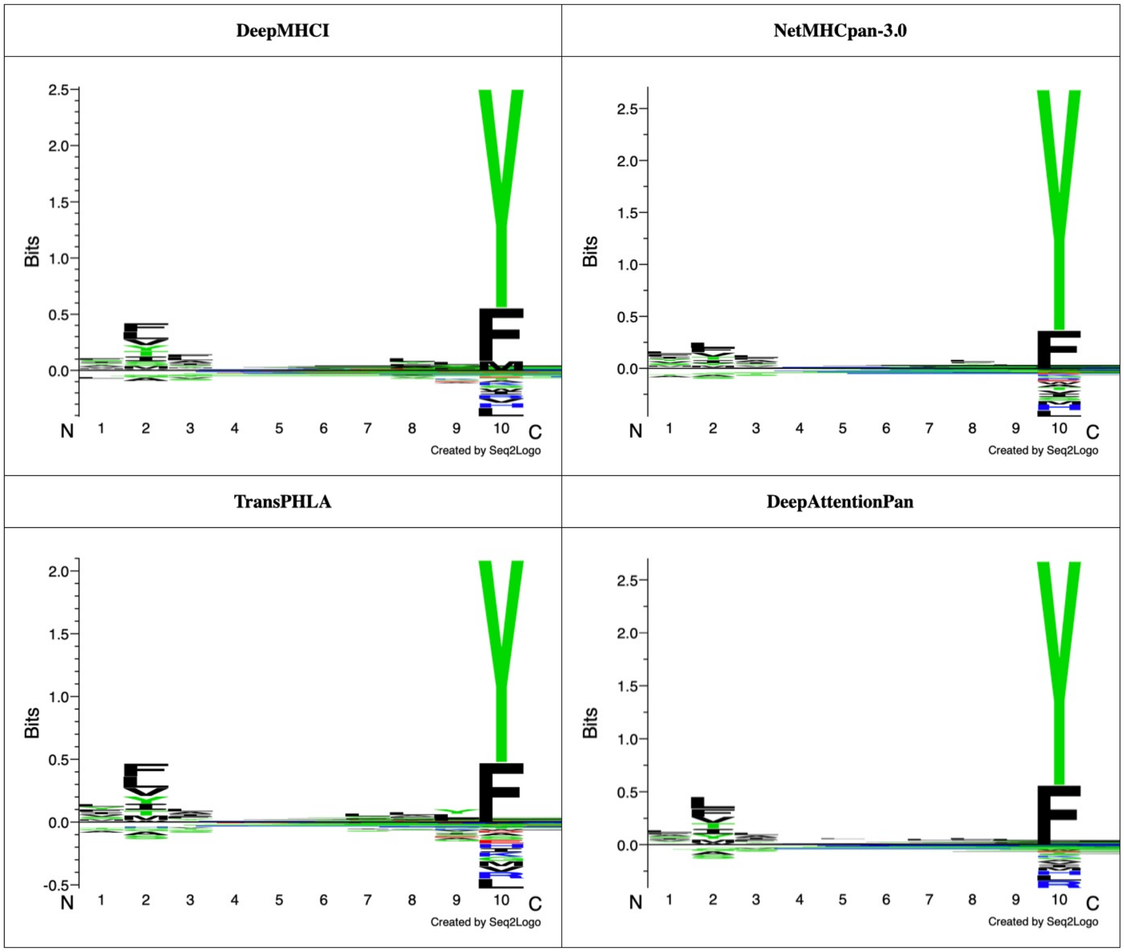

Figure S4: Motifs of DeepMHCI, NetMHCpan-3.0, TransPHLA and DeepAttentionPan on HLA-A29:02 under 10-mer.

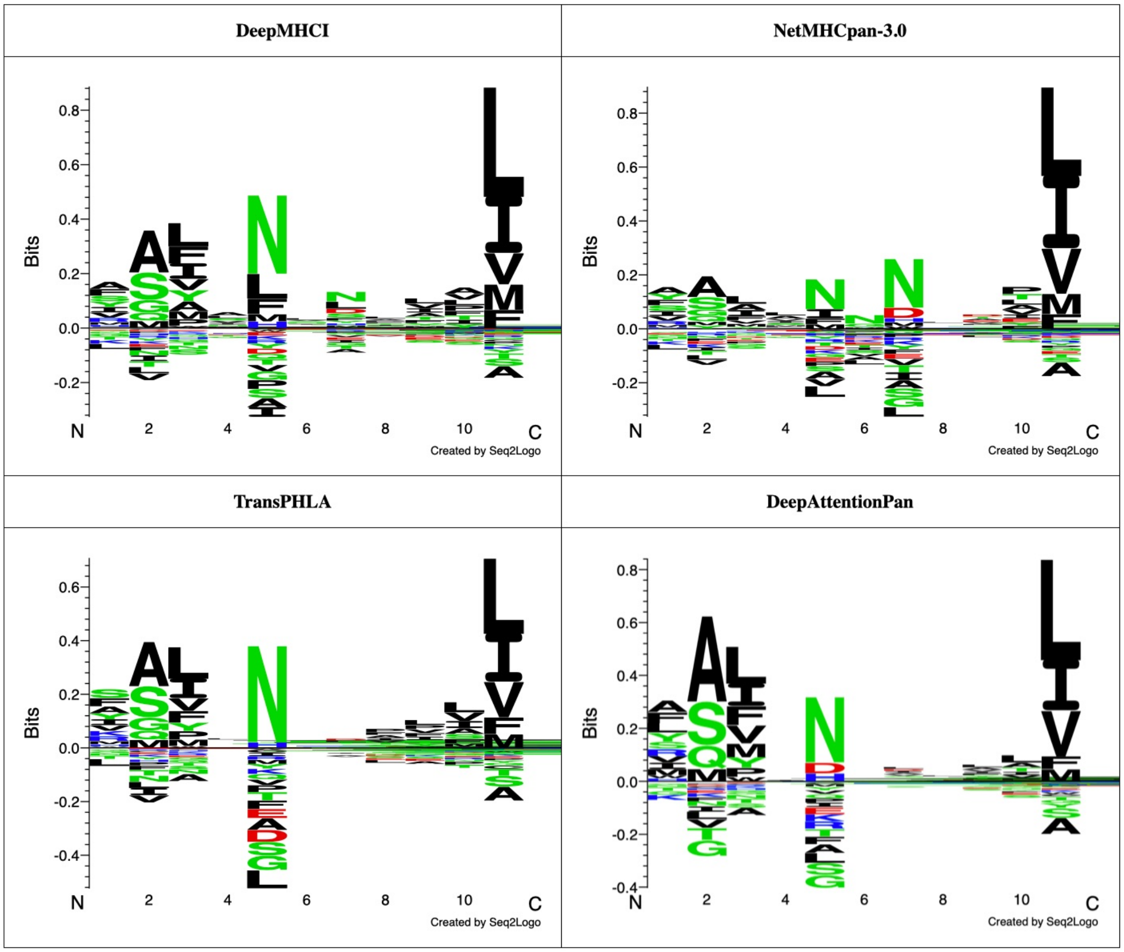

Figure S5: Motifs of DeepMHCI, NetMHCpan-3.0, TransPHLA and DeepAttentionPan on H-2-Db under 11-mer.

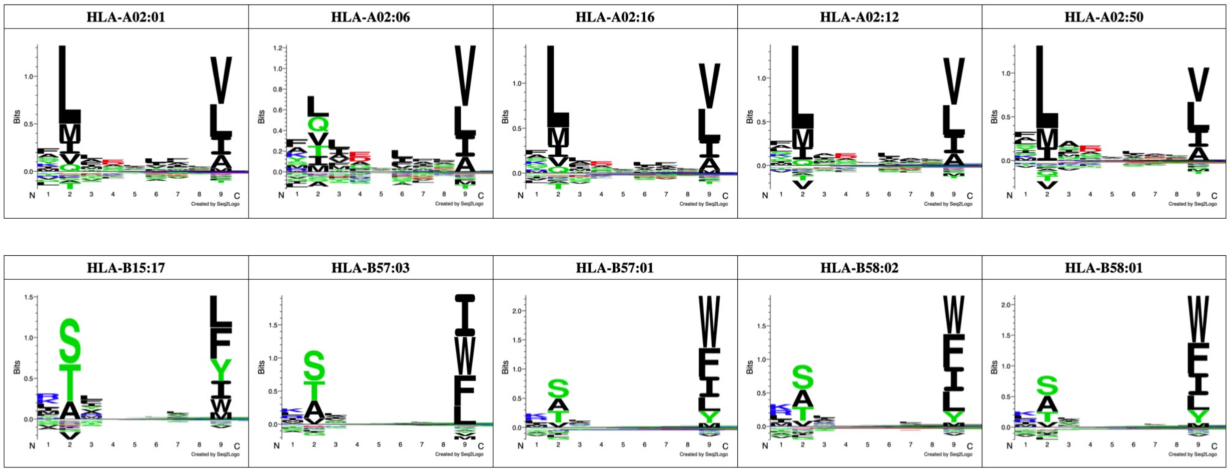

Figure S6: Motifs of molecules from HLA supertypes A2 and B58 generated by DeepMHCI.

Table S3: Detailed five-fold cross-validation performance of DeepMHCI and competing methods under 8mer.

allele	total	positive	NetMHCpan-3.0*		DeepLigand-BA		DeepAttentionPan		TransPHLA		DeepMHCI	
			AUC	PCC	AUC	PCC	AUC	PCC	AUC	PCC	AUC	PCC
H-2-Db	1113	31	0.761	0.466	0.743	0.393	0.756	0.368	0.783	0.484	**0.829**	**0.539**
H-2-Dd	48	5	**0.795**	0.648	0.749	**0.696**	0.781	0.686	0.693	0.521	0.763	0.600
H-2-Kb	1695	854	0.924	0.844	0.895	0.785	0.923	0.843	0.929	0.855	**0.935**	**0.871**
H-2-Kd	80	19	**0.795**	0.496	0.761	**0.545**	0.735	0.393	0.745	0.409	0.771	0.415
H-2-Kk	95	46	0.879	0.773	0.799	0.624	0.869	0.757	0.874	0.773	**0.898**	**0.808**
HLA-A02:01	167	18	**0.903**	0.794	0.899	0.768	0.878	0.719	0.868	0.744	0.887	**0.803**
HLA-A02:02	128	16	0.960	0.801	0.958	0.773	0.948	0.772	0.940	0.782	**0.969**	**0.819**
HLA-A02:03	131	20	**0.908**	0.801	0.907	0.805	0.896	0.816	0.894	0.803	0.899	**0.844**
HLA-A02:06	135	7	0.778	0.703	**0.837**	0.675	0.776	0.681	0.770	0.743	0.790	**0.765**
HLA-A03:01	63	3	0.947	**0.614**	0.900	0.576	**0.967**	0.571	0.939	0.532	0.950	0.611
HLA-A11:01	59	5	**0.996**	**0.883**	0.885	0.633	0.970	0.684	0.978	0.794	0.993	0.874
HLA-A23:01	57	7	**0.960**	0.746	0.911	0.679	0.943	0.771	0.854	0.742	0.943	**0.775**
HLA-A24:02	84	13	0.917	0.669	0.862	0.554	0.895	0.623	0.900	0.649	**0.924**	**0.706**
HLA-A30:02	53	4	0.827	**0.829**	0.811	0.393	0.837	0.774	**0.862**	0.753	0.776	0.774
HLA-A31:01	46	6	**0.888**	0.618	0.621	0.190	0.729	0.231	0.758	0.375	0.846	**0.635**
HLA-A68:02	128	5	0.930	**0.736**	0.777	0.620	0.810	0.686	0.846	0.610	**0.948**	0.726
HLA-B07:02	89	17	0.923	0.763	0.917	0.761	**0.943**	0.818	0.941	0.817	0.924	**0.825**
HLA-B08:01	52	14	0.962	0.829	**0.992**	**0.850**	0.991	0.803	0.972	0.810	0.987	0.818
HLA-B15:01	39	5	**0.718**	0.590	0.671	0.495	0.676	0.605	0.624	0.569	0.665	**0.648**
HLA-B15:03	29	4	0.710	0.595	0.720	0.468	0.800	0.472	0.840	0.571	**0.900**	**0.670**
HLA-B18:01	62	11	0.897	0.802	**0.939**	0.791	0.916	0.808	0.930	**0.823**	0.904	0.816
HLA-B27:05	281	54	0.897	0.780	0.864	0.716	0.906	0.801	0.933	0.846	**0.948**	**0.853**
HLA-B35:01	76	6	0.986	0.811	0.940	0.771	0.979	**0.865**	**1.000**	0.863	0.993	0.794
HLA-B40:01	59	3	**0.881**	**0.616**	0.679	0.372	0.857	0.597	0.863	0.610	0.851	0.575
HLA-B40:02	61	10	0.904	0.714	0.912	0.725	0.916	0.705	**0.941**	**0.756**	0.914	0.668
HLA-B44:02	62	5	0.930	0.616	0.944	0.648	0.944	0.650	0.958	0.663	**0.972**	**0.741**
HLA-B44:03	62	5	0.954	0.715	0.944	0.673	0.958	0.730	0.975	0.733	**0.986**	**0.779**
HLA-B45:01	63	4	0.987	**0.868**	0.979	0.757	**0.996**	0.765	0.992	0.819	0.992	0.842
HLA-B51:01	72	6	0.886	0.570	0.846	0.567	0.866	0.620	0.899	**0.666**	**0.902**	0.654
HLA-B57:01	45	4	0.896	0.640	**0.909**	0.461	0.909	0.593	0.909	**0.643**	0.909	0.613
HLA-B58:01	48	5	**0.874**	**0.789**	0.693	0.538	0.847	0.626	0.851	0.713	0.842	0.673
HLA-C06:02	25	3	**1.000**	**0.933**	1.000	0.919	1.000	0.876	1.000	0.884	1.000	0.916
Mamu-A01	532	171	0.926	0.844	0.906	0.790	0.916	0.832	0.923	0.849	**0.929**	**0.860**
Mamu-A02	229	69	0.913	0.800	0.892	0.733	**0.920**	0.804	0.917	**0.806**	0.917	0.805
Mamu-A07	113	17	**0.918**	**0.779**	0.842	0.626	0.841	0.670	0.909	0.722	0.914	0.770
Mamu-A11	224	46	0.902	0.749	0.847	0.618	0.885	0.709	**0.909**	0.769	0.909	**0.792**
Mamu-B01	166	7	**0.902**	0.537	0.877	0.456	0.821	0.455	0.893	**0.654**	0.868	0.620
Mamu-B03	241	58	0.917	0.846	0.919	0.817	0.923	0.843	0.920	0.852	**0.930**	**0.864**
Mamu-B08	240	49	0.920	0.850	0.903	0.788	0.930	0.851	**0.937**	**0.870**	0.933	0.865
Mamu-B17	167	6	0.975	0.704	0.940	0.496	0.867	0.332	0.942	0.521	**0.989**	**0.710**
Mamu-B3901	66	12	0.759	0.516	0.715	0.444	0.613	0.347	0.728	0.430	**0.815**	**0.620**
Mamu-B52	268	177	0.943	0.867	0.939	0.852	0.928	0.851	0.936	0.852	**0.944**	**0.868**
Average			0.896	0.727	0.858	0.639	0.878	0.676	0.888	0.707	**0.904**	**0.743**

Table S4: Detailed five-fold cross-validation performance of DeepMHCI and competing methods under 9mer.

allele	total	positive	NetMHCpan-3.0*		DeepLigand-BA		DeepAttentionPan		TransPHLA		DeepMHCI	
			AUC	PCC	AUC	PCC	AUC	PCC	AUC	PCC	AUC	PCC
BoLA-AW10	166	8	0.792	0.381	0.745	0.351	**0.929**	0.429	0.741	0.315	0.833	**0.479**
BoLA-D18.4	258	182	0.747	0.551	0.781	0.550	0.762	0.576	0.785	0.579	**0.806**	**0.639**
BoLA-HD6	267	218	0.732	0.513	0.703	0.402	0.788	0.561	0.788	0.575	**0.796**	**0.582**
BoLA-JSP.1	157	32	**0.620**	**0.271**	0.562	0.098	0.612	0.246	0.602	0.226	0.617	0.258
BoLA-T2C	90	84	0.813	0.455	0.688	0.163	**0.851**	**0.519**	0.772	0.466	0.762	0.509
BoLA-T2a	167	47	0.812	0.635	0.818	0.639	0.812	0.628	0.829	0.659	**0.837**	**0.680**
BoLA-T2b	157	38	0.816	0.566	0.704	0.397	0.778	0.565	**0.841**	**0.660**	0.834	0.648
H-2-Db	1798	614	0.929	0.845	0.889	0.767	0.926	0.834	0.932	0.847	**0.938**	**0.860**
H-2-Dd	249	17	0.804	0.582	0.802	0.554	0.829	0.611	**0.848**	0.585	0.825	**0.653**
H-2-Kb	1674	430	0.860	0.736	0.835	0.675	0.858	0.732	0.857	0.735	**0.871**	**0.756**
H-2-Kd	459	192	0.873	0.736	0.863	0.706	0.871	0.725	0.881	0.752	**0.884**	**0.782**
H-2-Kk	168	79	0.841	0.694	0.779	0.565	0.856	0.699	0.859	0.727	**0.868**	**0.749**
H-2-Ld	179	39	0.956	**0.815**	0.925	0.713	0.934	0.773	0.955	0.783	**0.964**	0.813
HLA-A01:01	3893	538	0.960	0.830	0.953	0.806	0.961	0.836	0.962	0.839	**0.969**	**0.852**
HLA-A02:01	9037	3269	**0.961**	0.881	0.954	0.866	0.959	0.877	0.960	0.879	0.961	**0.885**
HLA-A02:02	2464	1118	0.930	0.852	0.922	0.835	0.929	0.846	0.933	0.859	**0.935**	**0.862**
HLA-A02:03	4423	1542	0.958	0.883	0.951	0.870	0.955	0.878	0.957	0.881	**0.960**	**0.889**
HLA-A02:05	42	31	0.997	0.928	0.994	0.914	0.979	0.909	0.994	0.913	**1.000**	**0.943**
HLA-A02:06	3729	1521	0.915	0.812	0.904	0.795	0.915	0.814	**0.919**	0.820	0.917	**0.821**
HLA-A02:07	42	7	0.918	0.878	0.931	0.868	**0.935**	0.882	0.935	**0.885**	0.935	0.826
HLA-A02:11	1078	400	0.952	0.872	0.952	0.871	0.953	0.880	0.954	0.873	**0.956**	**0.881**
HLA-A02:12	1179	306	**0.965**	0.892	0.958	0.873	0.962	0.889	0.961	0.882	0.965	**0.896**
HLA-A02:16	916	178	0.967	0.866	0.970	0.866	0.968	0.879	0.966	0.864	**0.971**	**0.888**
HLA-A02:17	170	89	0.823	0.638	0.797	0.562	0.827	0.661	**0.860**	**0.697**	0.857	0.675
HLA-A02:19	1240	231	**0.981**	0.874	0.973	0.852	0.976	0.866	0.978	0.863	0.980	**0.882**
HLA-A02:50	134	88	0.981	0.928	0.978	0.916	**0.982**	**0.939**	0.978	0.920	0.982	0.934
HLA-A03:01	5477	1377	0.947	0.831	0.942	0.817	0.947	0.830	0.948	0.836	**0.951**	**0.845**
HLA-A03:19	30	14	0.857	0.609	0.808	0.476	0.839	0.558	**0.902**	**0.725**	0.848	0.631
HLA-A11:01	4536	1361	0.960	0.866	0.954	0.854	0.958	0.867	0.959	0.868	**0.961**	**0.876**
HLA-A23:01	1911	409	**0.921**	**0.768**	0.910	0.749	0.913	0.748	0.919	0.761	0.917	0.759
HLA-A24:02	2389	495	**0.915**	**0.778**	0.902	0.743	0.910	0.763	0.912	0.770	0.915	0.777
HLA-A24:03	1370	330	0.971	0.893	0.970	0.899	0.969	0.900	0.971	0.889	**0.972**	**0.904**
HLA-A25:01	934	71	0.994	0.839	0.987	0.817	0.992	0.850	0.992	0.833	**0.995**	**0.878**
HLA-A26:01	3758	408	0.953	0.819	0.942	0.775	0.951	0.818	0.953	0.824	**0.957**	**0.841**
HLA-A26:02	615	167	0.974	0.897	0.961	0.866	0.969	0.885	0.974	0.899	**0.976**	**0.916**
HLA-A26:03	516	61	0.962	0.794	0.928	0.755	0.955	0.775	**0.967**	0.810	0.960	**0.818**
HLA-A29:02	2103	531	0.887	0.764	0.882	0.753	0.888	0.772	0.892	0.765	**0.894**	**0.783**
HLA-A30:01	2561	735	0.940	0.838	0.932	0.825	0.938	0.843	0.940	0.845	**0.943**	**0.857**
HLA-A30:02	1324	362	0.847	0.684	0.840	0.654	0.855	0.695	0.850	0.685	**0.857**	**0.700**
HLA-A31:01	3939	1015	0.947	0.842	0.939	0.818	0.947	0.841	0.945	0.841	**0.949**	**0.849**
HLA-A32:01	829	395	0.900	0.800	0.893	0.770	0.903	0.797	0.909	0.809	**0.914**	**0.825**
HLA-A32:07	88	79	0.758	0.533	0.547	0.394	0.744	0.473	0.761	0.536	**0.782**	**0.606**
HLA-A32:15	74	59	0.776	**0.605**	0.668	0.443	**0.798**	0.560	0.747	0.552	0.769	0.596
HLA-A33:01	1927	387	0.946	0.817	0.934	0.782	0.945	0.808	0.948	0.824	**0.949**	**0.827**
HLA-A66:01	191	12	0.946	0.520	0.887	0.424	0.929	0.499	**0.965**	**0.597**	0.946	0.558
HLA-A68:01	2034	829	0.927	0.840	0.906	0.810	0.924	0.830	0.925	0.840	**0.928**	**0.847**
HLA-A68:02	3669	833	0.946	0.840	0.924	0.795	0.941	0.832	0.949	0.847	**0.950**	**0.858**
HLA-A68:23	81	76	0.705	0.451	0.626	0.318	0.697	0.440	0.737	0.476	**0.766**	**0.528**
HLA-A69:01	2553	248	0.953	0.798	0.947	0.776	0.954	0.805	**0.957**	0.796	0.957	**0.823**
HLA-A80:01	1164	122	0.965	0.800	0.959	0.779	0.966	0.809	0.965	0.806	**0.973**	**0.843**
HLA-B07:02	3862	1060	0.965	0.870	0.958	0.851	0.965	0.873	0.967	0.877	**0.969**	**0.883**
HLA-B08:01	3023	715	0.942	0.808	0.933	0.791	0.936	0.803	0.941	0.811	**0.947**	**0.828**
HLA-B08:02	997	37	0.991	0.821	0.986	0.825	0.993	0.852	0.993	0.829	**0.994**	**0.887**
HLA-B08:03	450	18	0.934	0.669	0.949	0.774	0.932	0.736	0.947	0.690	**0.955**	**0.786**
HLA-B14:01	40	16	**0.781**	**0.590**	0.698	0.455	0.716	0.452	0.716	0.502	0.745	0.542
HLA-B14:02	278	42	**0.910**	0.648	0.887	0.581	0.904	0.665	0.883	0.642	0.908	**0.674**
HLA-B15:01	4094	1184	0.932	0.779	0.926	0.765	0.931	0.778	0.932	0.783	**0.936**	**0.794**
HLA-B15:02	164	124	0.827	0.643	0.779	0.542	0.835	0.641	0.809	0.610	**0.844**	**0.677**
HLA-B15:03	581	364	0.929	0.853	0.926	0.843	0.933	0.861	**0.937**	**0.866**	0.935	0.864
HLA-B15:09	812	45	0.952	0.724	0.943	0.724	0.958	**0.766**	**0.960**	0.754	0.948	0.723
HLA-B15:17	1428	364	0.959	0.862	0.958	0.866	0.957	0.871	0.961	0.868	**0.962**	**0.879**
HLA-B15:42	362	3	**0.911**	**0.120**	0.413	-0.005	0.858	0.102	0.675	0.098	0.894	0.114
HLA-B18:01	2310	230	0.920	0.755	0.913	0.737	0.926	0.767	0.918	0.765	**0.929**	**0.783**
HLA-B27:05	2806	443	0.964	0.847	0.963	0.846	0.965	0.859	0.966	0.858	**0.967**	**0.867**
HLA-B35:01	2510	820	0.945	0.847	0.822	0.498	**0.948**	0.853	0.943	0.846	0.947	**0.857**
HLA-B35:03	151	15	**0.980**	**0.896**	0.937	0.834	0.969	0.863	0.976	0.860	0.978	0.896
HLA-B37:01	25	7	0.873	0.656	**0.956**	**0.814**	0.897	0.608	0.905	0.648	0.873	0.596
HLA-B38:01	490	147	0.992	0.924	0.944	0.743	**0.993**	0.923	0.991	0.926	0.992	**0.931**
HLA-B39:01	1756	301	0.969	0.843	**0.992**	**0.915**	0.963	0.843	0.962	0.831	0.970	0.859
HLA-B40:01	2819	478	0.978	0.876	0.959	0.830	0.979	0.887	0.979	0.882	**0.982**	**0.890**
HLA-B40:02	572	185	0.938	0.843	**0.974**	**0.874**	0.938	0.837	0.940	0.844	0.941	0.850
HLA-B40:13	59	53	0.726	0.445	**0.922**	**0.817**	0.780	0.546	0.657	0.510	0.667	0.433
HLA-B42:01	131	47	0.962	**0.911**	0.651	0.243	**0.964**	0.900	0.955	0.903	0.957	0.901
HLA-B44:02	1677	236	**0.968**	0.787	0.945	**0.890**	0.962	0.789	0.965	0.801	0.967	0.807
HLA-B44:03	733	207	0.957	0.875	0.959	0.761	0.956	0.878	0.954	0.876	**0.964**	**0.894**

HLA-B45:01	574	108	0.962	0.880	0.951	0.854	0.960	0.884	0.964	0.882	**0.969**	**0.888**
HLA-B45:06	360	4	0.916	0.164	**0.954**	**0.856**	0.825	0.110	0.927	0.215	0.835	0.143
HLA-B46:01	1785	94	0.971	0.730	0.542	0.055	0.967	0.763	**0.980**	0.764	0.978	**0.812**
HLA-B48:01	881	73	0.980	0.834	0.962	0.723	0.970	0.832	**0.981**	0.832	0.978	**0.855**
HLA-B51:01	2236	233	0.944	0.735	**0.981**	**0.862**	0.944	0.743	0.945	0.743	0.945	0.749
HLA-B53:01	1051	332	0.940	0.809	0.939	0.708	0.947	0.833	0.939	0.814	**0.948**	**0.834**
HLA-B54:01	721	145	0.933	0.844	0.927	0.785	0.936	**0.855**	**0.938**	0.849	0.937	0.854
HLA-B57:01	2523	383	0.976	0.865	0.927	0.825	0.975	0.869	0.976	0.871	**0.977**	**0.885**
HLA-B57:03	31	25	0.927	0.796	**0.973**	**0.863**	0.920	0.745	0.953	0.817	0.960	0.820
HLA-B58:01	2973	650	0.972	0.886	0.933	0.736	0.971	0.891	0.972	0.889	**0.976**	**0.906**
HLA-B58:02	54	9	0.859	0.648	**0.965**	**0.871**	0.775	0.540	0.854	0.668	0.877	0.682
HLA-B73:01	122	17	0.802	0.580	0.684	0.493	0.793	0.560	0.812	0.531	**0.832**	**0.592**
HLA-B81:01	26	13	**1.000**	0.919	0.799	0.566	1.000	0.886	0.994	0.880	0.994	**0.928**
HLA-B83:01	337	40	**0.957**	0.760	0.953	**0.816**	0.947	0.769	0.938	0.759	0.955	0.813
HLA-C03:03	153	115	0.845	0.719	**0.946**	**0.799**	0.876	0.761	0.870	0.743	0.874	0.756
HLA-C04:01	520	21	0.887	0.283	0.841	**0.695**	**0.912**	0.375	0.827	0.308	0.873	0.458
HLA-C05:01	171	68	0.953	0.859	0.884	0.279	0.937	0.834	**0.961**	0.851	0.959	**0.870**
HLA-C06:02	300	96	0.914	0.790	**0.936**	**0.829**	0.876	0.735	0.910	0.781	0.935	0.828
HLA-C07:01	219	87	0.872	0.697	0.878	**0.721**	0.841	0.636	0.858	0.673	**0.890**	0.717
HLA-C07:02	139	82	**0.887**	0.610	0.808	0.558	0.869	0.622	0.866	0.601	0.883	**0.652**
HLA-C08:02	87	32	0.786	0.555	0.831	0.496	**0.852**	0.634	0.766	0.538	0.826	**0.647**
HLA-C12:03	172	155	0.569	0.220	0.633	0.279	**0.683**	**0.293**	0.628	0.290	0.625	0.290
HLA-C14:02	235	185	0.768	0.567	0.512	0.101	**0.826**	**0.632**	0.759	0.583	0.793	0.595
HLA-C15:02	252	116	0.881	0.697	0.787	0.557	0.884	0.698	0.872	0.696	**0.895**	**0.738**
HLA-E01:01	96	14	**0.889**	0.708	0.866	0.674	0.820	0.607	0.874	0.720	0.889	**0.751**
HLA-E01:03	67	5	0.823	0.567	0.796	0.557	0.813	0.380	0.713	**0.632**	**0.874**	0.585
Mamu-A01	909	492	0.916	0.819	0.794	0.338	0.913	0.810	0.918	0.818	**0.922**	**0.828**
Mamu-A02	485	263	0.915	0.829	0.901	0.792	0.918	0.835	**0.928**	**0.843**	0.923	0.840
Mamu-A07	361	173	**0.983**	0.901	0.907	0.816	0.969	0.887	0.979	**0.905**	0.978	0.905
Mamu-A11	525	191	0.913	0.778	**0.971**	**0.874**	0.908	0.787	0.908	0.789	0.914	0.804
Mamu-A20102	138	26	0.880	0.803	0.885	0.743	0.882	**0.813**	**0.891**	0.798	0.842	0.782
Mamu-A2201	457	191	**0.960**	0.906	0.828	0.780	0.959	0.904	0.958	0.903	0.960	**0.911**
Mamu-A2601	180	49	0.783	0.566	**0.945**	**0.883**	0.732	0.508	0.815	0.622	0.814	0.665
Mamu-A70103	102	22	0.744	0.489	0.796	0.583	0.754	0.533	0.777	0.606	**0.808**	**0.616**
Mamu-B01	273	74	0.958	0.917	0.673	0.423	0.960	0.914	**0.963**	**0.919**	0.953	0.917
Mamu-B03	406	118	0.948	0.861	0.948	**0.898**	0.944	0.851	0.954	0.864	**0.955**	0.872
Mamu-B08	402	126	0.951	0.870	0.924	0.831	0.945	0.854	0.950	0.874	**0.955**	**0.887**
Mamu-B1001	140	42	0.817	0.652	**0.929**	**0.832**	0.812	0.694	0.838	0.686	0.819	0.658
Mamu-B17	721	280	0.904	0.818	0.783	0.599	0.882	0.775	0.900	0.817	**0.913**	**0.838**
Mamu-B3901	354	201	0.908	0.759	0.865	0.764	0.882	0.730	0.905	0.748	**0.910**	**0.774**
Mamu-B52	401	262	0.925	0.805	0.881	0.703	0.914	0.806	0.929	0.825	**0.935**	**0.831**
Mamu-B6601	100	72	0.800	0.637	**0.905**	**0.761**	0.794	0.612	0.813	0.639	0.830	0.676
Mamu-B8301	206	90	0.929	0.863	0.788	0.559	0.935	0.859	0.925	0.842	**0.937**	**0.867**
Mamu-B8701	119	42	0.788	0.590	**0.922**	**0.833**	0.797	0.630	0.855	0.736	0.904	0.797
Patr-A0101	239	54	0.851	0.712	0.745	0.476	0.832	0.655	**0.881**	**0.746**	0.847	0.676
Patr-A0301	204	27	0.925	0.774	0.825	0.691	0.911	0.754	0.915	0.789	**0.930**	**0.800**
Patr-A0401	178	40	0.905	0.825	0.910	0.755	0.892	0.804	**0.924**	**0.853**	0.908	0.853
Patr-A0701	318	67	0.845	0.690	**0.894**	**0.817**	0.853	0.657	0.871	0.685	0.856	0.663
Patr-A0901	207	76	**0.842**	0.699	0.833	0.634	0.813	0.690	0.834	0.738	0.842	**0.741**
Patr-B0101	484	112	0.935	0.847	0.804	0.688	0.941	0.846	**0.947**	**0.874**	0.939	0.861
Patr-B1301	129	75	0.924	**0.837**	**0.925**	0.807	0.911	0.820	0.919	0.819	0.919	0.832
Patr-B2401	193	62	0.959	0.852	0.902	0.792	0.966	**0.880**	**0.969**	0.863	0.965	0.856
SLA-10401	185	124	0.839	0.623	**0.957**	**0.808**	0.806	0.630	0.848	0.669	0.854	0.661
SLA-10701	23	14	0.548	-0.168	**0.776**	**0.476**	0.714	0.223	0.611	0.095	0.619	-0.031
SLA-20401	105	34	0.723	0.317	0.524	-0.055	0.760	0.412	0.761	0.374	**0.780**	**0.435**
SLA-30401	76	29	0.784	0.512	0.613	0.118	0.746	0.438	**0.794**	**0.531**	0.766	0.458
Average			0.896	0.726	0.865	0.679	0.895	0.726	0.896	0.737	**0.904**	**0.752**

Table S5: Detailed five-fold cross-validation performance of DeepMHCI and competing methods under 10mer.

allele	total	positive	NetMHCpan-3.0*		DeepLigand-BA		DeepAttentionPan		TransPHLA		DeepMHCI	
			AUC	PCC	AUC	PCC	AUC	PCC	AUC	PCC	AUC	PCC
H-2-Db	652	72	0.868	0.719	0.815	0.583	0.849	0.689	0.866	0.695	**0.885**	**0.724**
H-2-Dd	113	25	0.995	0.895	0.995	0.885	0.996	0.905	0.994	0.902	**0.997**	**0.906**
H-2-Kb	448	38	0.822	0.522	0.806	0.449	0.795	0.513	0.823	0.539	**0.841**	**0.543**
H-2-Kd	202	52	0.769	0.503	0.743	0.459	**0.783**	0.530	0.783	0.541	0.776	**0.542**
H-2-Kk	57	26	0.897	0.662	0.747	0.361	0.866	0.581	0.880	0.715	**0.921**	**0.763**
H-2-Ld	40	6	**0.902**	**0.684**	0.819	0.437	0.789	0.508	0.863	0.608	0.887	0.582
HLA-A01:01	699	138	0.910	0.762	0.886	0.724	0.907	0.760	0.914	0.785	**0.930**	**0.804**
HLA-A02:01	2749	1148	0.928	0.820	0.910	0.783	0.928	0.816	0.930	0.824	**0.935**	**0.834**
HLA-A02:02	1443	657	0.905	0.791	0.881	0.747	0.900	0.786	0.902	0.791	**0.910**	**0.808**
HLA-A02:03	1612	810	0.900	0.807	0.870	0.761	0.899	0.805	0.896	0.802	**0.910**	**0.825**
HLA-A02:05	25	19	0.965	0.858	0.974	0.884	**0.982**	0.859	0.974	0.878	0.982	**0.909**
HLA-A02:06	1621	678	0.906	0.795	0.888	0.759	0.901	0.784	0.908	0.794	**0.911**	**0.804**
HLA-A02:07	37	15	0.964	**0.898**	**0.982**	0.838	0.976	0.879	0.970	0.874	0.915	0.832
HLA-A02:17	176	88	**0.755**	0.525	0.639	0.372	0.711	0.459	0.715	0.492	0.750	**0.548**
HLA-A03:01	1691	719	0.905	0.812	0.888	0.765	0.907	0.806	0.913	0.818	**0.915**	**0.829**
HLA-A11:01	1677	754	0.914	0.810	0.898	0.780	0.917	0.808	0.919	0.819	**0.922**	**0.825**
HLA-A23:01	580	184	0.946	0.900	0.934	0.874	0.949	0.900	0.942	0.893	**0.953**	**0.911**
HLA-A24:02	689	189	0.946	0.866	0.927	0.833	0.945	0.864	0.938	0.851	**0.951**	**0.879**
HLA-A26:01	632	100	0.900	0.742	0.858	0.661	0.897	0.734	0.899	0.760	**0.905**	**0.773**
HLA-A29:02	522	171	0.863	0.735	0.844	0.714	0.865	0.743	0.862	0.735	**0.881**	**0.766**
HLA-A30:01	289	37	0.769	0.611	0.696	0.520	0.708	0.518	0.761	0.603	**0.799**	**0.655**
HLA-A30:02	596	226	**0.867**	**0.708**	0.829	0.661	0.850	0.688	0.833	0.651	0.862	0.704
HLA-A31:01	1636	575	**0.887**	0.746	0.864	0.707	0.883	0.746	0.882	0.739	0.887	**0.751**
HLA-A32:01	258	15	0.743	0.546	**0.772**	0.505	0.739	0.521	0.730	0.534	0.765	**0.596**
HLA-A33:01	1557	313	0.912	0.791	0.905	0.765	0.916	0.789	**0.924**	0.808	0.923	**0.813**
HLA-A68:01	1648	738	0.915	0.814	0.887	0.766	0.908	0.806	0.909	0.812	**0.918**	**0.827**
HLA-A68:02	1615	480	0.918	0.789	0.879	0.723	0.914	0.790	0.921	0.798	**0.931**	**0.814**
HLA-B07:02	570	237	0.910	0.818	0.873	0.759	0.911	0.824	0.915	0.821	**0.918**	**0.828**
HLA-B08:01	365	67	0.881	0.736	0.830	0.700	0.839	0.714	0.851	0.741	**0.902**	**0.806**
HLA-B15:01	252	164	0.953	0.857	0.960	0.852	0.977	0.888	0.977	0.891	**0.984**	**0.904**
HLA-B15:03	57	8	0.952	0.828	**0.980**	**0.850**	0.969	0.850	0.967	0.846	0.959	0.819
HLA-B18:01	354	51	0.901	0.765	0.829	0.627	0.888	0.745	0.892	0.742	**0.911**	**0.788**
HLA-B27:05	317	98	0.950	0.870	0.951	0.844	0.964	0.866	**0.967**	**0.881**	0.962	0.878
HLA-B35:01	522	136	0.879	0.792	0.865	0.733	**0.888**	0.788	0.879	0.782	0.885	**0.798**
HLA-B35:03	69	3	0.924	0.808	**0.970**	**0.844**	0.934	0.758	0.924	0.808	0.919	0.800
HLA-B40:01	495	149	0.883	0.752	0.871	0.731	0.878	0.748	**0.893**	**0.778**	0.891	0.778
HLA-B40:02	361	136	**0.910**	**0.835**	0.877	0.781	0.898	0.833	0.900	0.828	0.901	0.833
HLA-B42:01	56	11	**0.895**	0.788	0.883	0.801	0.873	0.828	0.828	0.781	0.875	**0.829**
HLA-B44:02	503	185	0.903	0.797	0.875	0.751	0.906	0.793	0.908	0.802	**0.924**	**0.826**
HLA-B44:03	516	192	0.894	0.807	0.880	0.769	0.903	0.813	0.898	0.810	**0.910**	**0.828**
HLA-B45:01	363	72	**0.860**	**0.756**	0.824	0.707	0.846	0.736	0.855	0.755	0.853	0.743
HLA-B51:01	498	54	**0.921**	0.699	0.899	0.665	0.918	0.695	0.907	**0.714**	0.917	0.687
HLA-B53:01	506	158	0.883	0.781	0.877	0.740	0.902	0.798	0.900	0.783	**0.905**	**0.802**
HLA-B54:01	343	67	0.944	0.787	0.940	0.803	0.946	0.802	**0.952**	**0.827**	0.951	0.815
HLA-B57:01	250	61	**0.767**	0.650	0.760	0.618	0.758	0.634	0.739	0.642	0.762	**0.672**
HLA-B58:01	241	72	0.795	0.681	0.779	0.636	0.794	0.677	0.813	0.712	**0.824**	**0.719**
Mamu-A01	681	274	0.869	0.720	0.873	0.714	0.869	0.716	**0.879**	**0.738**	0.878	0.734
Mamu-A02	333	153	**0.902**	**0.780**	0.849	0.691	0.884	0.748	0.884	0.752	0.894	0.775
Mamu-A07	109	13	0.923	**0.738**	0.883	0.612	0.911	0.696	0.909	0.721	**0.924**	0.738
Mamu-A11	287	116	0.895	0.802	0.865	0.739	0.885	0.783	0.902	0.801	**0.909**	**0.809**
Mamu-A2201	217	23	0.899	0.760	0.870	0.700	0.894	**0.770**	0.894	0.745	**0.903**	0.746
Mamu-B03	258	77	0.941	**0.878**	0.910	0.829	0.944	0.871	**0.948**	0.874	0.948	0.875
Mamu-B08	256	81	0.942	**0.873**	0.936	0.834	**0.955**	0.860	0.949	0.863	0.950	0.865
Mamu-B17	557	140	0.880	0.744	0.829	0.658	0.842	0.673	0.862	0.724	**0.883**	**0.756**
Mamu-B52	115	52	**0.861**	0.727	0.807	0.600	0.769	0.632	0.800	0.655	0.841	**0.728**
Mamu-B8301	217	129	**0.874**	0.746	0.823	0.687	0.858	0.728	0.874	**0.760**	0.868	0.760
Patr-A0101	184	54	**0.904**	0.795	0.863	0.704	0.873	0.748	0.900	**0.807**	0.868	0.739
Patr-A0301	158	30	0.932	**0.783**	0.909	0.706	0.912	0.713	**0.936**	0.783	0.935	0.775
Patr-A0401	140	34	0.896	0.800	0.877	0.755	0.884	0.777	**0.908**	**0.833**	0.885	0.780
Patr-A0701	262	64	0.836	0.661	0.782	0.569	0.811	0.622	0.824	0.667	**0.879**	**0.722**
Patr-A0901	195	76	0.848	**0.696**	0.817	0.629	0.859	0.680	0.808	0.668	**0.866**	0.693
Patr-B0101	226	56	0.919	0.814	0.881	0.710	0.912	0.797	0.929	0.817	**0.939**	**0.850**
Patr-B1301	71	51	0.922	**0.841**	0.894	0.785	**0.927**	0.840	0.895	0.811	0.922	0.801
Patr-B2401	130	22	**0.849**	**0.632**	0.771	0.513	0.770	0.546	0.824	0.600	0.783	0.591
Average			0.892	0.761	0.864	0.702	0.882	0.743	0.887	0.760	**0.897**	**0.773**

Table S6: Detailed five-fold cross-validation performance of DeepMHCI and competing methods under 11mer.

allele	total	positive	NetMHCpan-3.0*		DeepLigand-BA		DeepAttentionPan		TransPHLA		DeepMHCI	
			AUC	PCC	AUC	PCC	AUC	PCC	AUC	PCC	AUC	PCC
H-2-Db	212	14	0.847	0.556	0.848	0.389	0.826	0.556	0.847	0.576	**0.885**	**0.595**
H-2-Kb	160	17	**0.890**	**0.563**	0.756	0.309	0.770	0.442	0.715	0.345	0.875	0.556
H-2-Kd	102	32	0.841	**0.580**	0.792	0.446	0.849	0.576	0.838	0.554	**0.863**	0.558
H-2-Kk	51	25	0.778	0.530	0.528	0.013	0.718	0.406	0.774	0.476	**0.855**	**0.598**
HLA-A01:01	90	7	0.902	0.674	0.738	0.442	0.735	0.434	0.823	0.593	**0.909**	**0.688**
HLA-A02:01	139	32	**0.905**	0.767	0.821	0.628	0.898	0.740	0.893	**0.784**	0.900	0.777
HLA-A02:02	75	21	**0.902**	**0.778**	0.759	0.485	0.894	0.706	0.887	0.673	0.900	0.747
HLA-A02:03	88	22	**0.866**	**0.778**	0.705	0.551	0.798	0.690	0.798	0.685	0.861	0.766
HLA-A02:06	92	20	**0.839**	0.591	0.736	0.506	0.817	0.610	0.796	0.626	0.831	**0.668**
HLA-A03:01	73	14	0.889	0.714	0.873	0.521	**0.916**	**0.730**	0.880	0.653	0.884	0.680
HLA-A11:01	86	21	0.906	0.804	0.817	0.683	0.909	**0.812**	0.908	0.783	**0.919**	0.787
HLA-A23:01	60	5	**0.836**	**0.736**	0.749	0.552	0.749	0.476	0.775	0.562	0.789	0.723
HLA-A24:02	82	6	**0.904**	**0.610**	0.855	0.543	0.871	0.465	0.851	0.554	0.822	0.559
HLA-A26:01	58	3	0.703	0.412	**0.915**	**0.521**	0.770	0.410	0.848	0.446	0.782	0.444
HLA-A29:02	70	11	**0.992**	**0.801**	0.861	0.616	0.960	0.709	0.966	0.706	0.961	0.771
HLA-A30:02	51	13	**0.953**	**0.776**	0.836	0.600	0.901	0.671	0.895	0.693	0.937	0.775
HLA-A31:01	54	9	**0.970**	**0.766**	0.810	0.393	0.852	0.593	0.901	0.628	0.965	0.753
HLA-A33:01	36	3	0.949	0.853	0.960	0.583	0.970	0.554	**1.000**	0.793	0.970	**0.900**
HLA-A68:01	36	5	**0.994**	**0.873**	0.923	0.604	0.897	0.609	0.948	0.717	0.994	0.866
HLA-A68:02	85	7	0.949	**0.738**	0.689	0.404	0.934	0.731	0.934	0.628	**0.956**	0.733
HLA-B07:02	78	5	0.937	0.719	**0.995**	0.719	0.945	0.727	0.953	0.705	0.937	**0.748**
HLA-B08:01	65	3	0.478	0.333	0.446	0.096	0.398	0.266	0.554	0.313	**0.597**	**0.516**
HLA-B15:01	34	7	0.693	0.410	**0.746**	0.417	0.677	0.341	0.725	**0.505**	0.688	0.441
HLA-B18:01	71	16	0.790	0.566	0.842	0.512	0.857	0.576	0.920	0.731	**0.944**	**0.801**
HLA-B27:05	238	65	0.874	0.788	0.878	0.722	0.884	0.775	0.896	0.799	**0.912**	**0.831**
HLA-B35:01	63	9	**0.975**	**0.757**	0.887	0.660	0.879	0.658	0.922	0.674	0.914	0.738
HLA-B40:02	53	3	**1.000**	**0.822**	0.940	0.733	0.980	0.791	0.967	0.736	0.987	0.787
HLA-B44:02	44	9	0.965	0.830	0.943	0.699	0.994	**0.875**	0.956	0.783	**1.000**	0.821
HLA-B44:03	64	8	**0.958**	**0.820**	0.864	0.638	0.938	0.774	0.915	0.767	0.949	0.781
HLA-B45:01	50	6	**0.860**	**0.695**	0.742	0.587	0.769	0.555	0.803	0.582	0.833	0.646
HLA-B51:01	57	3	0.926	**0.709**	0.870	0.432	0.877	0.686	0.951	0.674	**0.988**	0.687
HLA-B57:01	51	18	**0.872**	0.732	0.833	0.642	0.865	**0.754**	0.867	0.725	0.845	0.715
HLA-B58:01	50	12	0.993	0.895	0.963	0.786	0.998	0.907	**1.000**	0.906	1.000	**0.924**
Mamu-A01	466	166	0.868	0.741	0.812	0.642	0.853	0.717	**0.875**	0.738	0.867	**0.743**
Mamu-A02	306	94	**0.833**	0.668	0.792	0.549	0.823	0.652	0.826	0.661	0.833	**0.684**
Mamu-A07	96	11	0.948	**0.826**	0.888	0.692	0.954	0.771	0.963	0.806	**0.967**	0.812
Mamu-A11	222	46	0.886	**0.721**	0.862	0.571	0.873	0.653	0.866	0.683	**0.894**	0.710
Mamu-B03	221	53	0.913	0.819	0.857	0.748	**0.924**	**0.835**	0.919	0.823	0.915	0.835
Mamu-B08	220	48	0.940	0.835	0.872	0.735	0.939	0.839	**0.951**	0.834	0.941	**0.859**
Mamu-B17	200	13	0.943	0.733	0.912	0.638	0.910	0.657	0.938	0.715	**0.944**	**0.744**
Mamu-B52	105	55	0.839	**0.672**	0.670	0.345	0.835	0.542	0.793	0.528	**0.878**	0.643
Patr-A0901	240	127	0.863	0.813	0.809	0.684	0.839	0.761	0.851	0.790	**0.892**	**0.821**
Patr-B0101	66	21	0.901	**0.788**	0.742	0.514	0.812	0.686	0.896	0.733	**0.905**	0.763
Average			0.885	0.712	0.817	0.548	0.857	0.645	0.874	0.667	**0.895**	**0.721**

Table S7: Detailed five-fold cross-validation performance of DeepMHCI and competing methods under ≥ 12 mer.

allele	total	positive	NetMHCpan-3.0*		DeepLigand-BA		DeepAttentionPan		TransPHLA		DeepMHCI	
			AUC	PCC	AUC	PCC	AUC	PCC	AUC	PCC	AUC	PCC
H-2-Db	30	3	0.926	0.618	0.914	0.516	0.951	0.589	0.938	0.519	**0.975**	**0.632**
H-2-Ld	91	71	0.812	0.656	0.770	0.509	0.847	0.607	**0.875**	0.703	0.873	**0.708**
HLA-A02:01	50	11	0.807	0.609	0.706	0.430	0.730	0.396	0.744	0.439	**0.821**	**0.629**
HLA-A02:02	42	11	0.880	**0.779**	0.774	0.594	0.812	0.580	0.806	0.626	**0.886**	0.765
HLA-A02:03	42	7	**0.902**	**0.739**	0.747	0.514	0.788	0.605	0.771	0.511	0.882	0.735
HLA-A02:06	45	4	0.732	**0.734**	0.683	0.474	0.720	0.684	0.628	0.533	**0.774**	0.712
HLA-A03:01	40	6	0.539	0.178	0.623	0.056	0.618	0.084	**0.696**	**0.201**	0.603	0.194
HLA-A68:02	43	3	**1.000**	0.784	0.483	0.117	0.700	0.458	0.817	0.449	0.925	**0.802**
HLA-B07:02	36	3	0.980	**0.806**	0.960	0.403	0.838	0.529	0.939	0.592	**0.990**	0.743
HLA-B08:01	29	4	0.750	0.426	0.770	0.395	**0.800**	0.287	0.670	0.118	0.790	**0.587**
HLA-B27:05	29	5	**0.825**	0.743	0.650	0.557	0.675	0.717	0.683	0.651	0.775	**0.820**
Average			0.832	0.643	0.734	0.415	0.771	0.503	0.779	0.486	**0.845**	**0.666**

Table S8: Detailed results of DeepMHCI and competing methods on ID2022.

IEDB reference	allele	length	total	pos	NetMHCpan-3.0 SRCC	NetMHCpan-3.0 AUC	DeepAttentionPan SRCC	DeepAttentionPan AUC	DeepLigand SRCC	DeepLigand AUC	TransPHLA SRCC	TransPHLA AUC	DeepMHCI SRCC	DeepMHCI AUC
1033576	HLA-A02:01	9	191	13	0.116	0.633	0.114	0.631	**0.12**	**0.637**	0.11	0.626	0.096	0.61
1034610	HLA-B53:01	10	12	8	0.857	0.944	**0.951**	1	0.874	0.969	0.895	0.938	0.923	**1**
315301	HLA-B39:09	9	17	10	0.781	0.957	0.854	1	0.854	1	**0.854**	**1**	0.83	0.986
1034596	HLA-A02:01	9	10	8	0.087	0.562	0.174	0.625	0	0.5	**0.174**	**0.625**	0.087	0.563
1035477	HLA-A30:01	9	18	3	0.445	0.844	0.417	0.822	**0.618**	**0.978**	0.388	0.8	0.445	0.844
1035477	HLA-A30:03	9	18	13	0.514	0.831	0.514	0.831	0.394	0.754	0.657	0.923	**0.681**	**0.938**
1036593	HLA-A02:01	9	14	9	0.462	0.778	**0.499**	**0.8**	0.314	0.689	0.462	0.778	0.462	0.778
1036593	HLA-A03:01	10	10	7	**0.478**	**0.786**	0.418	0.762	0.342	0.714	0.266	0.667	0.418	0.762
1036593	HLA-A11:01	10	11	6	0.751	0.933	0.751	0.933	0.462	0.767	0.693	0.9	**0.751**	**0.933**
1036593	HLA-B07:02	9	19	9	0.481	0.778	0.616	0.856	0.558	0.822	0.597	0.844	**0.712**	**0.911**
1036593	HLA-B08:01	9	12	8	0.768	0.969	0.717	0.938	0.358	0.719	0.615	0.875	**0.819**	**1**
1035424	H-2-Kb	9	13	8	0.127	0.575	0.254	0.65	0.169	0.6	0.38	0.725	**0.38**	**0.725**
1037140	HLA-A02:01	9	12	3	0.634	0.852	**0.711**	0.889	0.648	0.852	0.62	**0.926**	0.644	0.889
1037014	HLA-A02:01	9	16	11	0.659	0.909	0.629	0.891	0.687	0.927	0.687	0.927	**0.687**	**0.927**
1037480	HLA-E0101	9	142	56	0.6	0.854	0.575	0.84	0.589	0.848	**0.633**	**0.874**	0.61	0.86
1037480	HLA-E0101	9	11	4	0.553	0.964	0.507	0.929	0.402	0.893	0.603	1	**0.658**	**1**
1037812	HLA-A11:01	9	22	9	0.898	0.983	0.896	0.991	0.919	0.991	**0.939**	**0.991**	0.911	0.983
1037485	H-2-Db	9	12	2	0.648	1	0.648	1	0.648	1	0.583	0.95	**0.648**	**1**
1037485	H-2-Kb	8	11	6	0.866	1	0.693	0.9	0.635	0.867	0.866	1	**0.866**	**1**
1037485	H-2-Kb	9	12	8	0.154	0.594	0.154	0.594	0.154	0.594	0.102	0.563	**0.205**	**0.625**
1037895	HLA-A02:01	9	12	8	0.497	0.656	0.727	0.719	0.434	0.469	**0.867**	**0.844**	0.797	0.781
1037895	HLA-A02:01	10	11	6	0.7	0.833	0.873	**0.9**	0.518	0.767	**0.882**	0.867	0.836	0.833
1038478	HLA-A02:01	9	25	12	0.855	0.994	0.855	0.994	0.833	0.981	0.833	0.981	**0.855**	**0.994**
1038307	HLA-A02:01	9	10	6	0.711	0.917	0.711	0.917	**0.782**	**0.958**	0.711	0.917	0.711	0.917
1038759	H-2-Kb	9	12	7	0.196	0.614	0.416	0.743	0.122	0.571	0.122	0.571	**0.465**	**0.771**
1037887	H-2-Kd	9	38	16	0.793	0.952	0.864	**0.991**	0.764	0.926	0.884	0.989	**0.89**	0.983
1037887	H-2-Kd	10	34	11	0.288	0.518	0.413	0.601	0.372	0.585	**0.593**	**0.704**	0.52	0.64
1037887	H-2-Kd	11	38	11	0.614	0.815	0.741	0.845	0.606	0.724	**0.82**	**0.909**	0.78	0.872
1039262	HLA-B08:01	9	11	2	0.671	1	**0.671**	**1**	0.596	0.944	0.596	0.944	0.596	0.944
1039262	HLA-B15:01	9	11	7	0.418	0.75	0.478	0.786	0.598	0.857	**0.777**	**0.964**	0.657	0.893
1039262	HLA-B35:01	9	14	11	0.41	0.788	**0.54**	**0.879**	0.108	0.576	0.281	0.697	0.281	0.697
1039262	HLA-B39:01	9	10	4	0.853	1	0.853	1	0.711	0.917	0.853	1	**0.853**	**1**
1039262	HLA-B52:01	9	12	3	**0.307**	**0.704**	0.251	0.667	0.251	0.667	0.139	0.593	0.251	0.667
1039262	HLA-C07:01	9	16	5	0.395	0.745	**0.6**	**0.873**	0.219	0.636	0.366	0.727	0.453	0.782
1039262	HLA-A02:01	9	28	2	0.343	0.885	0.343	0.885	0.292	0.827	**0.412**	**0.962**	0.378	0.923
1038983	HLA-A03:01	9	14	4	**0.67**	0.85	0.638	**0.9**	0.554	0.85	0.607	0.875	0.629	0.875
1038983	HLA-A03:01	10	13	3	0.707	0.933	0.713	1	0.622	1	0.699	0.967	**0.748**	**1**
1038983	HLA-A24:02	9	16	3	0.347	0.692	0.321	0.692	0.356	0.667	0.262	0.692	**0.371**	**0.692**
1039280	HLA-A02:01	9	41	23	**0.615**	**0.857**	0.457	0.766	0.482	0.78	0.552	0.821	0.519	0.802
1039423	HLA-A02:01	9	25	4	0.523	0.762	**0.691**	**0.881**	0.513	0.762	0.568	0.833	0.593	0.821
1039423	HLA-A24:02	10	15	8	0.536	0.875	0.575	0.875	**0.714**	0.893	0.639	**0.893**	0.582	0.875
1039423	HLA-B07:02	9	15	5	0.735	1	**0.836**	1	0.783	0.98	0.779	0.98	0.798	**1**
1039777	H-2-Db	9	10	3	0.821	1	0.679	1	0.601	1	0.847	1	**0.925**	**1**
1039777	H-2-Kb	8	14	4	**0.801**	**0.95**	0.599	0.85	0.608	0.8	0.737	0.9	0.762	0.925
1039777	H-2-Kb	9	10	2	0.83	1	0.782	1	0.721	0.875	0.745	1	**0.83**	**1**
1039793	HLA-A02:01	9	26	17	0.566	0.843	0.523	0.817	0.587	0.856	**0.598**	**0.863**	0.587	0.856
1039793	HLA-A02:03	9	20	15	0.551	0.867	0.531	0.853	0.571	0.88	0.531	0.853	**0.631**	**0.92**
1039793	HLA-A24:02	10	13	7	**0.784**	**0.952**	0.701	0.905	0.701	0.905	0.701	0.905	0.742	0.929
1039793	HLA-A33:03	9	21	16	0.037	0.525	**0.203**	**0.637**	0.092	0.562	0.111	0.575	0.148	0.6
1039793	HLA-A03:01	9	24	16	0.217	0.594	0.001	0.547	0.013	0.523	**0.237**	**0.633**	0.142	0.562
1040276	HLA-A02:01	9	21	14	**0.5**	**0.806**	0.317	0.694	0.234	0.643	0.217	0.633	0.417	0.755
1016643	HLA-A02:01	9	14	9	0.832	1	0.832	1	0.758	0.956	0.832	1	**0.832**	**1**
1038337	HLA-A26:01	10	11	2	0.149	0.611	0.224	0.667	0.075	0.556	0.149	0.611	**0.298**	**0.722**
1040281	HLA-A01:01	9	16	2	0.574	1	0.574	1	0.574	1	0.574	1	**0.574**	**1**
1040281	HLA-A02:01	9	16	10	0.392	0.733	0.392	0.733	**0.504**	**0.8**	0.392	0.733	0.448	0.767
1040281	HLA-A02:03	9	16	7	0.506	0.794	**0.533**	**0.81**	0.451	0.762	0.506	0.794	0.424	0.746
1040281	HLA-A03:01	9	16	4	0.47	0.812	0.595	0.896	0.219	0.646	**0.626**	**0.917**	0.501	0.833
1040281	HLA-A11:01	9	16	3	**0.295**	**0.718**	0.226	0.667	-0.017	0.487	0.052	0.539	-0.087	0.436
1040281	HLA-A23:01	9	16	6	**0.84**	**1**	0.812	0.983	0.812	0.983	0.756	0.95	0.784	0.967
1040281	HLA-A24:01	9	16	3	0.191	0.641	0.226	0.667	0.156	0.615	0.191	0.641	**0.226**	**0.667**
1040281	HLA-B15:01	9	16	7	0.478	0.778	**0.506**	**0.794**	0.478	0.778	0.369	0.714	0.424	0.746
1040281	HLA-B40:01	9	16	2	0.574	1	0.574	1	0.574	1	0.574	1	**0.574**	**1**
1032718	HLA-A02:01	10	10	2	0.522	0.875	0.522	0.875	**0.696**	**1**	0.435	0.813	0.522	0.875
1041542	HLA-A02:01	9	34	26	0.339	0.731	0.339	0.731	0.346	0.736	**0.346**	**0.736**	0.339	0.731
Average					0.536	0.830	0.552	0.843	0.480	0.794	0.545	0.836	**0.569**	**0.846**

Table S9: Detailed results of DeepMHCI† and DBTpred† on ID2022. Both methods were only used 5 models to the ensemble.

					DBTpred†		DeepMHCI †	
IEDB reference	allele	length	total	pos	SRCC	AUC	SRCC	AUC
1033576	HLA-A02:01	9	191	13	**0.146**	**0.667**	0.111	0.627
1034610	HLA-B53:01	10	12	8	0.769	0.781	**0.86**	**1**
315301	HLA-B39:09	9	17	10	0.756	0.943	**0.854**	**1**
1034596	HLA-A02:01	9	10	8	0.087	**0.563**	**0.087**	0.562
1035477	HLA-A30:01	9	18	3	**0.618**	**0.978**	0.417	0.822
1035477	HLA-A30:03	9	18	13	0.562	0.862	**0.705**	**0.954**
1036593	HLA-A02:01	9	14	9	0.24	0.644	**0.536**	**0.822**
1036593	HLA-A03:01	10	10	7	**0.494**	**0.81**	0.342	0.714
1036593	HLA-A11:01	10	11	6	**0.866**	**1**	0.751	0.933
1036593	HLA-B07:02	9	19	9	0.654	0.878	**0.674**	**0.889**
1036593	HLA-B08:01	9	12	8	0.41	0.75	**0.615**	**0.875**
1035424	H-2-Kb	9	13	8	-0.338	0.3	**0.507**	**0.8**
1037140	HLA-A02:01	9	12	3	**0.613**	0.889	0.609	**0.889**
1037014	HLA-A02:01	9	16	11	0.658	0.909	**0.658**	**0.909**
1037480	HLA-E0101	9	142	56	0.564	0.833	**0.604**	**0.857**
1037480	HLA-E0101	9	11	4	**0.858**	0.929	0.662	**1**
1037812	HLA-A11:01	9	22	9	0.823	0.974	**0.915**	**0.991**
1037485	H-2-Db	9	12	2	0.194	0.65	**0.648**	**1**
1037485	H-2-Kb	8	11	6	0.52	0.8	**0.866**	**1**
1037485	H-2-Kb	9	12	8	0.154	0.594	**0.154**	**0.594**
1037895	HLA-A02:01	9	12	8	**0.853**	**0.875**	0.811	0.781
1037895	HLA-A02:01	10	11	6	0.8	0.767	**0.845**	**0.833**
1038478	HLA-A02:01	9	25	12	0.844	0.987	**0.844**	**0.987**
1038307	HLA-A02:01	9	10	6	0.569	0.833	**0.782**	**0.958**
1038759	H-2-Kb	9	12	7	**0.514**	**0.8**	0.416	0.743
1037887	H-2-Kd	9	38	16	0.297	0.682	**0.892**	**0.986**
1037887	H-2-Kd	10	34	11	0.345	0.593	**0.531**	**0.636**
1037887	H-2-Kd	11	38	11	0.131	0.515	**0.788**	**0.875**
1039262	HLA-B08:01	9	11	2	0.596	0.944	**0.596**	**0.944**
1039262	HLA-B15:01	9	11	7	0.478	0.786	**0.657**	**0.893**
1039262	HLA-B35:01	9	14	11	-0.022	0.485	**0.194**	**0.636**
1039262	HLA-B39:01	9	10	4	0.142	0.583	**0.853**	**1**
1039262	HLA-B52:01	9	12	3	0.251	0.667	**0.251**	**0.667**
1039262	HLA-C07:01	9	16	5	0.249	0.655	**0.453**	**0.782**
1039262	HLA-A02:01	9	28	2	0.326	0.865	**0.361**	**0.904**
1038983	HLA-A03:01	9	14	4	0.594	0.85	**0.682**	**0.9**
1038983	HLA-A03:01	10	13	3	**0.823**	0.9	0.677	**1**
1038983	HLA-A24:02	9	16	3	0.309	0.59	**0.371**	**0.744**
1039280	HLA-A02:01	9	41	23	0.428	0.749	**0.498**	**0.79**
1039423	HLA-A02:01	9	25	4	0.535	0.714	**0.588**	**0.81**
1039423	HLA-A24:02	10	15	8	0.489	0.821	**0.525**	**0.857**
1039423	HLA-B07:02	9	15	5	0.791	**0.98**	**0.798**	0.96
1039777	H-2-Db	9	10	3	0.032	0.667	**0.925**	**1**
1039777	H-2-Kb	8	14	4	0.357	0.6	**0.689**	**0.9**
1039777	H-2-Kb	9	10	2	0.6	1	**0.83**	**1**
1039793	HLA-A02:01	9	26	17	0.404	0.745	**0.609**	**0.869**
1039793	HLA-A02:03	9	20	15	0.431	0.787	**0.611**	**0.907**
1039793	HLA-A24:02	10	13	7	0.619	0.857	**0.742**	**0.929**
1039793	HLA-A33:03	9	21	16	0.074	0.55	**0.074**	**0.55**
1039793	HLA-A03:01	9	24	16	**0.248**	**0.648**	0.134	0.547
1040276	HLA-A02:01	9	21	14	0.317	0.694	**0.4**	**0.745**
1016643	HLA-A02:01	9	14	9	0.795	0.978	**0.832**	**1**
1038337	HLA-A26:01	10	11	2	0.075	0.556	**0.447**	**0.833**
1040281	HLA-A01:01	9	16	2	0.574	1	**0.574**	**1**
1040281	HLA-A02:01	9	16	10	**0.476**	**0.783**	0.448	0.767
1040281	HLA-A02:03	9	16	7	**0.506**	**0.794**	0.478	0.778
1040281	HLA-A03:01	9	16	4	**0.626**	**0.917**	0.501	0.833
1040281	HLA-A11:01	9	16	3	**0.295**	**0.718**	-0.122	0.41
1040281	HLA-A23:01	9	16	6	0.784	0.967	**0.784**	**0.967**
1040281	HLA-A24:01	9	16	3	0.087	0.564	**0.156**	**0.615**
1040281	HLA-B15:01	9	16	7	0.369	0.714	**0.506**	**0.794**
1040281	HLA-B40:01	9	16	2	0.574	1	**0.574**	**1**
1032718	HLA-A02:01	10	10	2	**0.609**	**0.938**	0.522	0.875
1041542	HLA-A02:01	9	34	26	**0.346**	**0.736**	0.332	0.726
Average					0.456	0.775	**0.563**	**0.843**

Table S10: Detailed results of DeepMHCI and competing methods on HPV2019 under 8mer. Results of methods with † were only used 5 models to ensemble.

allele	DBTpred†	DeepMHCI†	NetMHCpan-3.0	DeepLigand-BA	DeepAttentionPan	TransPHLA	DeepMHCI
HLA-A02:01	**0.800**	0.725	0.738	0.525	0.650	0.575	0.725
HLA-A11:01	0.981	0.991	1.000	0.648	0.704	0.870	**1.000**
	**0.891**	0.858	0.869	0.587	0.677	0.723	0.863

Table S11: Detailed results of DeepMHCI and competing methods on HPV2019 under 9mer. Results of methods with † were only used 5 models to ensemble.

allele	DBTpred†	DeepMHCI†	NetMHCpan-3.0	DeepLigand-BA	DeepAttentionPan	TransPHLA	DeepMHCI
HLA-A02:01	0.845	0.911	**0.932**	0.905	0.917	0.908	0.911
HLA-A03:01	**0.947**	0.913	0.880	0.856	0.889	0.909	0.899
HLA-A11:01	0.943	**0.953**	0.900	0.871	0.946	0.935	0.939
HLA-A24:02	0.842	0.820	0.811	0.728	**0.858**	0.814	0.814
HLA-B15:01	0.842	0.865	**0.940**	0.910	0.914	0.853	0.910
	0.884	0.893	0.892	0.854	**0.905**	0.884	0.895

Table S12: Detailed results of DeepMHCI and competing methods on HPV2019 under 10mer. Results of methods with † were only used 5 models to ensemble.

allele	DBTpred†	DeepMHCI†	NetMHCpan-3.0	DeepLigand-BA	DeepAttentionPan	TransPHLA	DeepMHCI
HLA-A02:01	0.823	0.894	0.797	0.834	**0.906**	0.899	0.899
HLA-A03:01	0.934	0.964	0.949	0.929	0.969	0.969	**0.980**
HLA-A11:01	0.815	0.812	0.819	0.838	0.846	0.788	**0.873**
HLA-A24:02	0.600	0.714	0.761	0.693	0.701	**0.795**	0.737
HLA-B15:01	**0.939**	0.885	0.874	0.896	0.896	0.926	0.894
	0.822	0.854	0.840	0.838	0.864	0.876	**0.877**

Table S13: Detailed results of DeepMHCI and competing methods on HPV2019 under 11mer. Results of methods with † were only used 5 models to ensemble.

allele	DBTpred†	DeepMHCI†	NetMHCpan-3.0	DeepLigand-BA	DeepAttentionPan	TransPHLA	DeepMHCI
HLA-A02:01	0.714	**0.847**	0.799	0.744	0.744	0.752	0.835
HLA-A03:01	0.434	0.329	0.395	**0.434**	0.329	0.289	0.289
HLA-A11:01	0.674	0.800	0.745	0.283	0.542	0.655	**0.828**
HLA-A24:02	0.576	0.758	0.717	0.798	0.783	0.758	**0.854**
HLA-B15:01	0.800	0.770	0.790	0.700	0.590	0.850	**0.950**
	0.640	0.701	0.689	0.592	0.598	0.661	**0.751**

Table S14: Detailed results of DeepMHCI and competing methods on EP2017 with all lengths.

allele	total	NetMHCpan-3.0		DeepAttentionPan		TransPHLA		DeepMHCI	
		Frank	AUC	Frank	AUC	Frank	AUC	Frank	AUC
HLA-A01:01	60	**0.010295906**	**0.989684443**	0.0127798	0.987199807	0.012421246	0.987557375	0.011392573	0.988586136
HLA-A02:01	733	0.022819548	0.977102927	0.024010814	0.975906336	0.02444117	0.975474509	**0.022600783**	**0.977324019**
HLA-A02:02	2	**0.035195975**	**0.964767006**	0.038877119	0.961081986	0.038877119	0.961081986	0.035716808	0.96424563
HLA-A02:03	2	0.003159558	0.996835443	0.004608833	0.995381955	0.003159558	0.996835443	**0.002369668**	**0.997626582**
HLA-A02:05	4	0.032242792	0.967711608	0.033630457	0.966331745	0.040683057	0.959262295	**0.029402161**	**0.970558**
HLA-A02:06	7	0.050775526	0.949019756	0.043747486	0.95606606	**0.043008895**	**0.956826817**	0.060489044	0.939240922
HLA-A02:11	2	0.02030323	0.979641658	0.016234527	0.983721427	**0.010832784**	**0.989137799**	0.013535487	0.986427772
HLA-A02:17	1	0.120654397	0.879098361	0.132924335	0.866803279	0.096114519	0.903688525	**0.079754601**	**0.920081967**
HLA-A03:01	52	0.023855737	0.976038866	**0.02260679**	**0.977297019**	0.024563546	0.97532358	0.02639736	0.973489265
HLA-A11:01	143	0.018787465	0.981032979	0.019326661	0.980546661	0.019645131	0.980211479	**0.016492875**	**0.983384263**
HLA-A23:01	5	**0.003534746**	**0.996456877**	0.003749572	0.996241693	0.006568149	0.993416709	0.003749572	0.996241693
HLA-A24:02	105	**0.017342577**	**0.982603803**	0.018797782	0.981136827	0.01985807	0.980081148	0.020360678	0.979572878
HLA-A24:03	1	0.007731959	0.992248062	0.007731959	0.992248062	**0**	**1**	0.00257732	0.997416021
HLA-A25:01	4	0.004522463	0.99546906	0.037248946	0.962675781	0.005979568	0.994008847	**0.003653205**	**0.996341254**
HLA-A26:01	4	**0.039016713**	**0.960919524**	0.048879158	0.95104263	0.05179468	0.948120059	0.045102162	0.954820035
HLA-A29:02	22	0.009343259	0.990634398	**0.008008458**	**0.991972448**	0.009064307	0.990913711	0.008152496	0.991827751
HLA-A30:01	8	0.054024668	0.945655146	0.060020361	0.939483031	0.061966545	0.937532479	**0.04204456**	**0.957656957**
HLA-A30:02	7	0.015831368	0.984142152	0.01780306	0.982164004	**0.015825293**	**0.984147062**	0.018259165	0.981708183
HLA-A31:01	1	0.055648117	0.944333333	0.092635788	0.907333333	0.060313229	0.939666667	**0.04865045**	**0.951333333**
HLA-A32:01	8	0.013476074	0.986515215	**0.012965297**	**0.987028225**	0.030824539	0.969151632	0.019922893	0.980068896
HLA-A33:01	1	0.002350867	0.997648442	**0.002350867**	**0.997648442**	0.003232442	0.996766608	0.002644725	0.997354497
HLA-A68:01	21	0.020381157	0.979565938	0.052411601	0.947454677	0.030638983	0.9692806	**0.014033323**	**0.98591317**
HLA-A68:02	4	0.001754097	0.998245366	0.00143272	0.998566847	**0.000964349**	**0.999035364**	0.001359234	0.998640354
HLA-B07:02	136	0.0116687	0.98828641	0.011620964	0.988344645	0.012305049	0.987647268	**0.010925721**	**0.989029317**
HLA-B08:01	39	0.028507582	0.971424175	**0.025823202**	**0.974095865**	0.031166812	0.96875198	0.027067775	0.9728608
HLA-B08:02	3	**0.002042396**	**0.997953424**	0.002056395	0.997939367	0.00411279	0.995878735	0.002746526	0.997247804
HLA-B14:02	2	**0.000293858**	**0.999706055**	0.002188914	0.997809193	0.000881575	0.999118166	0.00217389	0.997822972
HLA-B15:01	13	**0.027972822**	**0.971975364**	0.033367146	0.96656858	0.036157043	0.963769032	0.045318035	0.954599224
HLA-B15:02	4	0.015958372	0.98401977	0.015700876	0.984272457	0.013688568	0.986289735	**0.012336074**	**0.987646645**
HLA-B15:03	4	0.073833548	0.925812961	**0.070740401**	**0.928966606**	0.073092585	0.92656247	0.075236526	0.924459117
HLA-B18:01	12	**0.005353092**	**0.994634687**	0.008564064	0.991409618	0.0072077	0.992774909	0.009887167	0.990088287
HLA-B27:05	17	0.015135093	0.984840117	0.010574466	0.989407493	**0.008992384**	**0.990993733**	0.018110999	0.981856703
HLA-B35:01	69	0.010027133	0.989951102	0.013498249	0.986465731	0.012284914	0.987680872	**0.007920103**	**0.992062284**
HLA-B35:03	5	**0.006136007**	**0.993854327**	0.008359944	0.991627592	0.009283891	0.9906998	0.006654892	0.993333146
HLA-B37:01	7	0.024851504	0.97512452	**0.014345543**	**0.985637465**	0.018952988	0.981009988	0.028909274	0.971057816
HLA-B38:01	3	**0.000783622**	**0.999216147**	0.002260245	0.99773785	0.001273386	0.998726239	0.002162292	0.997835832
HLA-B39:01	2	0.008196721	0.991758242	0.005464481	0.994505495	0.008196721	0.991758242	**0.005464481**	**0.994505495**
HLA-B39:06	1	0	1	0	1	0	1	**0**	**1**
HLA-B40:01	13	0.002440257	0.997557187	**0.002324496**	**0.997673028**	0.002969624	0.997027332	0.002437525	0.997559966
HLA-B40:02	10	0.006480757	0.993496526	0.005515865	0.994462774	**0.003840351**	**0.996147578**	0.005732105	0.994244806
HLA-B44:02	10	0.013571336	0.986419815	0.007552749	0.992442565	**0.007451838**	**0.992542794**	0.008020461	0.991974339
HLA-B44:03	9	**0.001452529**	**0.998545976**	0.008129656	0.991838777	0.005718496	0.994261843	0.002181932	0.997812073
HLA-B45:01	2	0.007925658	0.992061597	0.008150434	0.991836167	**0.005025126**	**0.994966443**	0.0096007	0.990383744
HLA-B46:01	3	**0.037568567**	**0.962391034**	0.098104563	0.901789857	0.071310116	0.928610847	0.083498108	0.916411496
HLA-B51:01	27	0.018037126	0.981911894	0.025448529	0.974495535	**0.008204639**	**0.991776455**	0.012029592	0.987940385
HLA-B53:01	10	0.011695964	0.988287765	**0.006197648**	**0.993794791**	0.006832434	0.993158605	0.00683492	0.99315573
HLA-B57:01	15	0.009730974	0.990255132	0.011326335	0.98865747	0.011088276	0.988897162	**0.008436099**	**0.99155253**
HLA-B58:01	22	0.016447361	0.983498415	**0.011855679**	**0.988109967**	0.012940915	0.987022444	0.012228728	0.987733903
HLA-C03:03	4	**0.006484558**	**0.993509234**	0.065795451	0.934176681	0.047777596	0.952203456	0.012677007	0.987316498
HLA-C05:01	3	0.047727784	0.952184442	0.039808194	0.960118532	0.039808194	0.960118532	**0.015924627**	**0.984046059**
HLA-C06:02	12	0.038379246	0.961438039	0.052175102	0.947530366	0.049029889	0.950714948	**0.028850527**	**0.971015405**
HLA-C07:02	6	**0.032760752**	**0.967169614**	0.040330234	0.959591364	0.033644279	0.966292922	0.035661218	0.964273032
Average		0.020471375	0.979474122	0.025385812	0.974550733	0.022192603	0.977748524	**0.019763239**	**0.98018625**

Table S15: Detailed results of DeepMHCI and competing methods on EP2017 under 8mer.

allele	total	NetMHCpan-3.0 Frank	DeepAttentionPan Frank	TransPHLA Frank	DeepMHCI Frank
HLA-A01:01	2	0.006311961	0.011016451	**0.004553467**	0.006607112
HLA-A02:01	7	**0.024359717**	0.063946755	0.053759232	0.035291451
HLA-A03:01	1	**0**	0.002028398	0.004056795	0.004056795
HLA-A11:01	5	0.008132473	0.04838558	0.020116779	**0.005752553**
HLA-A29:02	2	0.00399304	**0**	0.002564103	0.002710989
HLA-A30:02	1	0.023752969	0.042755344	**0.021377672**	0.023752969
HLA-B07:02	8	**0.007042497**	0.007966701	0.008174873	0.009037562
HLA-B08:01	6	**0.016547259**	0.017585487	0.017837521	0.021484293
HLA-B08:02	2	0.002028398	0.001014199	0.002028398	**0.001014199**
HLA-B18:01	3	0.00048962	0.00146886	**0.000293772**	0.000685468
HLA-B35:01	3	**0.002563914**	0.071406902	0.072303434	0.012476904
HLA-B37:01	2	0.056902782	0.027545138	**0.014492623**	0.056373132
HLA-B40:01	1	**0.006302521**	0.017507003	0.016106443	0.017507003
HLA-B40:02	1	0.026490066	0.026490066	**0.013245033**	0.026490066
HLA-B51:01	6	0.003948849	0.009567956	**0.002242174**	0.003577263
HLA-B58:01	2	0.005188266	**0.002355152**	0.004000657	0.003562828
HLA-C06:02	1	0.001175088	0.001762632	0.000881316	**0.000293772**
Average		**0.011484084**	0.020753096	0.015178488	0.01356908

Table S16: Detailed results of DeepMHCI and competing methods on EP2017 under 9mer.

allele	total	NetMHCpan-3.0 Frank	DeepAttentionPan Frank	TransPHLA Frank	DeepMHCI Frank
HLA-A01:01	38	**0.008594643**	0.011730877	0.008796164	0.009783205
HLA-A02:01	555	**0.021594662**	0.022981009	0.022994408	0.021863931
HLA-A02:02	2	**0.035195975**	0.038877119	0.038877119	0.035716808
HLA-A02:03	2	0.003159558	0.004608833	0.003159558	**0.002369668**
HLA-A02:05	3	0.023116858	0.029483789	0.030757175	**0.022039377**
HLA-A02:06	6	0.032418191	**0.02421881**	0.031019956	0.029382812
HLA-A02:11	1	0.016216216	0.018918919	**0.005405405**	0.010810811
HLA-A02:17	1	0.120654397	0.132924335	0.096114519	**0.079754601**
HLA-A03:01	24	**0.018807361**	0.018835795	0.024553922	0.024122704
HLA-A11:01	69	0.0175846	0.016840218	0.016689106	**0.01601339**
HLA-A23:01	5	**0.003534746**	0.003749572	0.006568149	0.003749572
HLA-A24:02	69	**0.017399307**	0.019150432	0.020610589	0.021029186
HLA-A25:01	2	**0.000587717**	0.001028504	0.000734646	0.000734646
HLA-A26:01	1	**0.133815552**	0.153707052	0.173598553	0.16636528
HLA-A29:02	17	**0.003956842**	0.004315417	0.00539085	0.004129628
HLA-A30:01	8	0.054024668	0.060020361	0.061966545	**0.04204456**
HLA-A30:02	5	0.016897857	**0.015342288**	0.016333484	0.018234917
HLA-A32:01	7	0.014351425	**0.011458113**	0.033884298	0.022097147
HLA-A33:01	1	0.002350867	**0.002350867**	0.003232442	0.002644725
HLA-A68:01	11	**0.009491648**	0.012896089	0.009625391	0.010865259
HLA-A68:02	3	0.001652925	0.001126441	**0.00069791**	0.001126441
HLA-B07:02	63	0.01504635	0.014234715	0.015588749	**0.013851294**
HLA-B08:01	31	0.024879027	**0.023435041**	0.027651292	0.024447917
HLA-B08:02	1	**0.002070393**	0.004140787	0.008281573	0.00621118
HLA-B14:02	2	**0.000293858**	0.002188914	0.000881575	0.00217389
HLA-B15:01	8	**0.007753548**	0.011658892	0.020343466	0.010419979
HLA-B15:02	4	0.015958372	0.015700876	0.013688568	**0.012336074**
HLA-B15:03	4	0.073833548	**0.070740401**	0.073092585	0.075236526
HLA-B18:01	6	**0.00418248**	0.005539701	0.004725368	0.005268256
HLA-B27:05	10	0.007578936	0.007386451	**0.005951451**	0.006011969
HLA-B35:01	44	0.012067434	0.010075611	0.011693158	**0.00981334**
HLA-B35:03	2	**0.011518348**	0.015314219	0.019681613	0.014873431
HLA-B37:01	4	0.015038742	**0.011332131**	0.024736583	0.022404663
HLA-B38:01	2	**0.001175433**	0.002350867	0.001910079	0.002203938
HLA-B39:01	2	0.008196721	0.005464481	0.008196721	**0.005464481**
HLA-B39:06	1	0	0	0	**0**
HLA-B40:01	8	0.001800454	**0.001139273**	0.002472927	0.001322934
HLA-B40:02	8	0.004716202	0.003583573	**0.003144809**	0.003853873
HLA-B44:02	3	0.008477479	**0.004336692**	0.005814907	0.005026823
HLA-B44:03	1	0.001175433	0.000881575	0.001469292	**0.000587717**
HLA-B45:01	1	0.013400335	0.015075377	**0.010050251**	0.016750419
HLA-B46:01	3	**0.037568567**	0.098104563	0.071310116	0.083498108
HLA-B51:01	4	0.009644491	0.005039236	**0.003319052**	0.006707811
HLA-B53:01	7	0.013495329	**0.007036462**	0.007773864	0.008327464
HLA-B57:01	13	0.01048188	0.009693048	0.009381886	**0.00818816**
HLA-B58:01	9	0.03729987	**0.022764369**	0.024373368	0.026515398
HLA-C05:01	2	0.05412844	0.016513761	0.016513761	**0.005504587**
HLA-C06:02	11	0.041761443	0.056758054	0.053407032	**0.031446596**
HLA-C07:02	4	0.019964274	0.007038179	0.006228279	**0.003366007**
Average		0.020590069	0.021471267	0.021687602	**0.01952432**

Table S17: Detailed results of DeepMHCI and competing methods on EP2017 under 10mer.

allele	total	NetMHCpan-3.0 Frank	DeepAttentionPan Frank	TransPHLA Frank	DeepMHCI Frank
HLA-A01:01	14	0.011853479	0.010390048	0.013639073	**0.009911409**
HLA-A02:01	158	0.027930855	0.025488453	0.027652354	**0.024558175**
HLA-A02:05	1	0.059620596	**0.046070461**	0.070460705	0.051490515
HLA-A02:06	1	0.16091954	0.16091954	**0.114942529**	0.247126437
HLA-A02:11	1	0.024390244	**0.013550136**	0.016260163	0.016260163
HLA-A03:01	24	0.031319317	0.028615469	**0.028026509**	0.031831985
HLA-A11:01	57	0.023899905	**0.018541636**	0.023766126	0.019758234
HLA-A24:02	30	0.018993577	**0.017498453**	0.01845712	0.019521044
HLA-A24:03	1	0.007731959	0.007731959	**0**	0.00257732
HLA-A25:01	1	0.000587889	0	**0**	0.002939447
HLA-A26:01	3	0.0074171	0.013936526	0.011193389	**0.004681122**
HLA-A29:02	3	0.043433101	0.034274662	**0.034214029**	0.034576418
HLA-A30:02	1	**0.00257732**	0.005154639	0.007731959	0.012886598
HLA-A31:01	1	0.055648117	0.092635788	0.060313229	**0.04865045**
HLA-A32:01	1	0.007348618	0.023515579	0.009406232	**0.004703116**
HLA-A68:01	4	0.019258827	0.017513006	0.019577984	**0.016860592**
HLA-A68:02	1	0.002057613	0.002351558	**0.001763668**	0.002057613
HLA-B07:02	49	0.010327871	0.011235854	0.01095036	**0.009772094**
HLA-B08:01	1	**0.008298755**	0.043568465	0.037344398	0.010373444
HLA-B15:01	5	**0.060323661**	0.068100353	0.061458766	0.101154924
HLA-B18:01	1	0.004081633	0.016326531	0.008163265	**0.004081633**
HLA-B27:05	2	0.022742818	0.009917921	**0.009404925**	0.013337893
HLA-B35:01	8	0.009129106	0.005486627	0.003466551	**0.002865395**
HLA-B35:03	2	0.00249853	0.003233392	**0.000881834**	0.001322751
HLA-B37:01	1	0	0	0.004739336	**0**
HLA-B38:01	1	0	0.002079002	**0**	0.002079002
HLA-B40:01	3	0.001712187	0.001003066	0.000905084	**0.000905084**
HLA-B40:02	1	0.000587889	0	0	**0**
HLA-B44:02	6	0.018380153	0.010419569	**0.009512276**	0.010854023
HLA-B44:03	5	0.002379465	0.002491254	0.003161827	**0.002100532**
HLA-B45:01	1	0.00245098	0.00122549	**0**	0.00245098
HLA-B51:01	6	0.0059678	0.008285558	**0.004196375**	0.005823304
HLA-B53:01	2	0.011099155	0.006066591	0.006953647	**0.004734442**
HLA-B57:01	2	**0.004850088**	0.021942699	0.022179808	0.010047702
HLA-B58:01	11	**0.001433326**	0.004657756	0.005212592	0.002115253
HLA-C03:03	2	**0.006497834**	0.121126291	0.086304565	0.021992669
HLA-C05:01	1	**0.034926471**	0.086397059	0.086397059	0.036764706
HLA-C07:02	2	**0.058353707**	0.106914343	0.088476279	0.100251641
Average		**0.02029025**	0.027596467	0.023871421	0.023511003

Table S18: Detailed results of DeepMHCI and competing methods on EP2017 under 11mer.

allele	total	NetMHCpan-3.0 Frank	DeepAttentionPan Frank	TransPHLA Frank	DeepMHCI Frank
HLA-A01:01	6	**0.018764214**	0.025586853	0.035161097	0.026636439
HLA-A02:01	13	**0.01216139**	0.028512604	0.031391859	0.023435438
HLA-A03:01	2	0.013560627	0.015513211	**0.005491892**	0.00651125
HLA-A11:01	10	0.007031317	0.007748168	**0.003517963**	0.009675693
HLA-A24:02	6	**0.008435182**	0.021238957	0.018208837	0.016870999
HLA-A25:01	1	0.016326531	0.146938776	0.02244898	**0.010204082**
HLA-A68:01	4	0.007225434	0.015818148	0.009057363	**0.000240848**
HLA-B07:02	14	0.005472679	0.004955251	0.006387777	**0.004255882**
HLA-B08:01	1	0.23296355	**0.131537242**	0.213946117	0.158478605
HLA-B18:01	1	0.033591731	**0.010335917**	0.049095607	0.072351421
HLA-B27:05	4	0.03329918	0.021516393	**0.017930328**	0.051741803
HLA-B35:01	12	0.0059664	0.01148908	**0.004353478**	0.004528855
HLA-B35:03	1	0.002646281	0.004704499	0.005292561	**0.000882094**
HLA-B40:01	1	0.005880623	0.000588062	**0**	0.000882094
HLA-B44:02	1	0	0	0	**0**
HLA-B44:03	1	0	0	0	**0**
HLA-B51:01	11	0.035356778	0.050893842	**0.015419795**	0.021960395
HLA-B53:01	1	0.000294031	0.000588062	**0**	0.000588062
HLA-C03:03	2	0.006471281	0.01046461	0.009250626	**0.003361345**
Average		0.023444591	0.026759457	0.023523909	**0.02171607**

Table S19: Detailed results of DeepMHCI and competing methods on EP2017 under 12mer.

allele	total	NetMHCpan-3.0 Frank	DeepAttentionPan Frank	TransPHLA Frank	DeepMHCI Frank
HLA-A03:01	1	0.010336779	0.003667889	**0.000333444**	0.01267089
HLA-A11:01	2	**0**	0.112727273	0.083636364	0.000909091
HLA-A68:01	2	0.108829569	0.412731006	0.211498973	**0.05338809**
HLA-B18:01	1	0	0.038461538	**0**	0.008547009
HLA-B27:05	1	0.002824859	**0**	0.002824859	0.014124294
HLA-B44:03	2	**0**	0.02991453	0.017094017	0.004273504
Average		0.020331868	0.099583706	0.05256461	**0.01565215**

Table S20: Detailed results of DeepMHCI and competing methods on EP2017 under 13mer.

allele	total	NetMHCpan-3.0 Frank	DeepAttentionPan Frank	TransPHLA Frank	DeepMHCI Frank
HLA-B07:02	2	0	0	**0**	0.00127551
HLA-B35:01	2	0.004291845	0.046034827	0.018137831	**0**
Average		0.002145923	0.023017413	0.009068916	**0.00063776**

Table S21: Performance of DeepMHCI with different kernel sizes over BD2017.

Method	AUC	PCC
$DeepMHCI_9$	0.883	0.727
$DeepMHCI_{11}$	0.885	0.729
$DeepMHCI_{13}$	0.886	0.726
DeepMHCI	**0.889**	**0.731**

Table S22: Performance of DeepMHCI with different embedding options over BD2017.

Method	AUC	PCC
BLOSUM50 matrix	0.840	0.623
ESM-1 pretrained (12layer)	0.847	0.652
Learned embedding	**0.866**	**0.676**

References

[1] O. Lund, M. Nielsen, C. Kesmir, A. G. Petersen, C. Lundegaard, P. Worning, C. Sylvester-Hvid, K. Lamberth, G. Røder, S. Justesen, et al. Definition of supertypes for HLA molecules using clustering of specificity matrices. *Immunogenetics*, 55:797–810, 2004.

[2] A. T. Nguyen, C. Szeto, and S. Gras. The pockets guide to HLA class I molecules. *Biochemical Society Transactions*, 49(5):2319–2331, 2021.

[3] A. Rives, J. Meier, T. Sercu, S. Goyal, Z. Lin, J. Liu, D. Guo, M. Ott, C. L. Zitnick, J. Ma, et al. Biological structure and function emerge from scaling unsupervised learning to 250 million protein sequences. *Proceedings of the National Academy of Sciences*, 118(15):e2016239118, 2021.

[4] A. Sette and J. Sidney. Nine major HLA class I supertypes account for the vast preponderance of HLA-A and-B polymorphism. *Immunogenetics*, 50(3-4):201–212, 1999.

[5] J. Sidney, B. Peters, N. Frahm, C. Brander, and A. Sette. HLA class I supertypes: a revised and updated classification. *BMC immunology*, 9(1):1–15, 2008.
